# Transition from C_3_ to proto-Kranz to C_3_–C_4_ intermediate type in the genus *Chenopodium* (Chenopodiaceae)

**DOI:** 10.1007/s10265-019-01135-5

**Published:** 2019-08-31

**Authors:** Yuki Yorimitsu, Aya Kadosono, Yuto Hatakeyama, Takayuki Yabiku, Osamu Ueno

**Affiliations:** 1 Graduate School of Bioresource and Bioenvironmental Sciences, Kyushu University, Motooka 744, Nishi-ku, Fukuoka, 819-0395 Japan; 2 School of Agriculture, Kyushu University, Motooka 744, Nishi-ku, Fukuoka, 819-0395 Japan; 3 Present Address: Faculty of Agriculture, Kyushu University, Motooka 744, Nishi-ku, Fukuoka, 819-0395 Japan

**Keywords:** C_3_–C_4_ intermediate plant, *Chenopodium*, CO_2_ compensation point, Glycine decarboxylase, Leaf anatomy, Proto-Kranz plant

## Abstract

The Chenopodiaceae is one of the families including C_4_ species among eudicots. In this family, the genus *Chenopodium* is considered to include only C_3_ species. However, we report here a transition from C_3_ photosynthesis to proto-Kranz to C_3_–C_4_ intermediate type in *Chenopodium*. We investigated leaf anatomical and photosynthetic traits of 15 species, of which 8 species showed non-Kranz anatomy and a CO_2_ compensation point (*Γ*) typical of C_3_ plants. However, 5 species showed proto-Kranz anatomy and a C_3_-like *Γ*, whereas *C. strictum* showed leaf anatomy and a *Γ* typical of C_3_–C_4_ intermediates. *Chenopodium album* accessions examined included both proto-Kranz and C_3_–C_4_ intermediate types, depending on locality. Glycine decarboxylase, a key photorespiratory enzyme that is involved in the decarboxylation of glycine, was located predominantly in the mesophyll (M) cells of C_3_ species, in both M and bundle-sheath (BS) cells in proto-Kranz species, and exclusively in BS cells in C_3_–C_4_ intermediate species. The M/BS tissue area ratio, number of chloroplasts and mitochondria per BS cell, distribution of these organelles to the centripetal region of BS cells, the degree of inner positioning (vacuolar side of chloroplasts) of mitochondria in M cells, and the size of BS mitochondria also changed with the change in glycine decarboxylase localization. All *Chenopodium* species examined were C_3_-like regarding activities and amounts of C_3_ and C_4_ photosynthetic enzymes and δ^13^C values, suggesting that these species perform photosynthesis without contribution of the C_4_ cycle. This study demonstrates that *Chenopodium* is not a C_3_ genus and is valuable for studying evolution of C_3_–C_4_ intermediates.

**Electronic supplementary material:**

The online version of this article (10.1007/s10265-019-01135-5) contains supplementary material, which is available to authorized users.

## Introduction

Photorespiration is an inevitable metabolic process in C_3_ plants that use ribulose 1,5-bisphosphate carboxylase/oxygenase (Rubisco) for primary fixation of CO_2_. In ordinary air, one-fourth of photosynthetically fixed CO_2_ was lost by photorespiration, resulting in decreased photosynthetic efficiency (Bauwe 2011; Sage et al. 2012). On the other hand, C_4_ plants have biochemical traits of photosynthesis associated with anatomical differentiation of leaves to reduce photorespiration (Hatch 1987). In general, C_4_ leaves exhibit Kranz-type anatomy, in which an external layer of mesophyll (M) and an internal layer of bundle sheath (BS) encircle vascular bundles (Edwards and Voznesenskaya 2011; Lundgren et al. 2014). In M cells, atmospheric CO_2_ is first fixed in C_4_ compounds, and they are moved to BS cells, where they are decarboxylated to supply CO_2_ for Rubisco. Increased CO_2_ concentration within BS cells suppresses photorespiration (Hatch 1987). Therefore, C_4_ plants have higher photosynthetic efficiency than C_3_ plants in environments that promote high rates of photorespiration. The C_4_ trait has evolved independently at least 66 times in flowering plants in response to multiple ecological drivers including decreasing atmospheric CO_2_ concentration (Sage et al. 2012). Much effort has recently focused on elucidating the evolution from C_3_ to C_4_ plants (reviewed in Christin and Osborne 2014; Sage et al. 2012, 2014). These works may provide a clue for engineering of C_4_ elements into C_3_ crops (Schlüter and Weber 2016).

An early study reported the existence of plants with traits intermediate between those of C_3_ and C_4_ plants (Kennedy and Laetsch 1974). These are called C_3_–C_4_ intermediate plants (Edwards and Ku 1987; Monson and Rawsthorne 2000). Leaves of most C_3_–C_4_ intermediate plants show Kranz-like anatomy, in which BS cells contain numerous chloroplasts and mitochondria (Edwards and Ku 1987; Sage et al. 2014). In these plants, the values of the CO_2_ compensation point (*Г*) and O_2_ inhibition of photosynthesis are intermediate between the values of C_3_ and C_4_ plants (Edwards and Ku 1987; Monson and Rawsthorne 2000). An apparent reduction in photorespiration in C_3_–C_4_ intermediates is accomplished by a particular biochemical system operating between the M and BS cells. This system is called the glycine shuttle (Monson and Rawsthorne 2000; Rawsthorne 1992). In C_3_–C_4_ intermediates, at least the P-protein, one of the 4 subunits constituting the glycine decarboxylase (GDC) multi-enzyme system, is absent in the M mitochondria, which renders GDC non-functional, and glycine generated in the M cells must be transported into the BS mitochondria to be decarboxylated by GDC (Rawsthorne 1992; Rawsthorne et al. 1988). In the BS cells of most C_3_–C_4_ intermediates, mitochondria are located between the centripetally located chloroplasts and the inner tangential walls (Brown and Hattersley 1989; Muhaidat et al. 2011; Rawsthorne 1992; Sage et al. 2013; Ueno et al. 2003; Ueno 2011). As a result, a large part of CO_2_ released from mitochondria by decarboxylation of glycine is captured by chloroplasts, resulting in suppression of CO_2_ loss from BS cells. Many C_3_–C_4_ intermediate species reduce photorespiratory CO_2_ loss only by using the glycine shuttle (type I intermediates), but in some intermediates a C_4_ cycle complements the glycine shuttle (type II intermediates; Edwards and Ku 1987).

Until now, 56 species with C_3_–C_4_ intermediate traits have been found in 2 monocot and 11 eudicot families (Lundgren and Christin 2017). Some genera, such as *Flaveria* (Ku et al. 1991; Sage et al. 2013), *Heliotropium* (Muhaidat et al. 2011; Vogan et al. 2007), *Salsola* (Voznesenskaya et al. 2013), *Eleocharis* (Roalson et al. 2010; Ueno et al. 1989), *Alloteropsis* (Bianconi et al. 2018), and *Neurachne* (Christin et al. 2012), include C_3_, C_3_–C_4_ intermediate, and C_4_ types and provide a unique opportunity to trace the evolution from C_3_ to C_4_ plants. Many of these studies suggest that the initial event in the evolution of C_3_–C_4_ and then C_4_ plants is the appearance of chloroplasts and mitochondria along the centripetal region of BS cells, with GDC activity present in both M and BS mitochondria. This phase is called the proto-Kranz type (Muhaidat et al. 2011; Sage et al. 2012, 2014). Type I intermediates would evolve from the proto-Kranz type with predominant accumulation of GDC in BS mitochondria and its decrease in M mitochondria. Complementation of the glycine shuttle by the increasing activity of the C_4_ cycle would then lead to type II intermediates. Finally, the C_4_ type would evolve from type II intermediates through the C_4_–like plants (Edwards and Ku 1987; Sage et al. 2012, 2014).

Among eudicots, the goosefoot family Chenopodiaceae (Caryophyllales) includes the greatest number of C_4_ species (about 40% of 1400 species; Sage et al. 1999). C_4_ species have been detected in four Chenopodiaceae subfamilies: Chenopodioideae, Salicornioideae, Salsoloideae, and Suaedoideae (Carolin et al. 1975; Jacobs 2001; Kadereit et al. 2003; Pyankov et al. 2001; Voznesenskaya et al. 2001b, 2002, 2007; Wen and Zhang 2011). In the Chenopodioideae, C_4_ species occur in the tribes Atripliceae (e.g., *Atriplex* and *Axyris*) and Camphorosmeae (e.g., *Bassia* and *Kochia*), but they have not been found in the tribe Chenopodieae (Freitag and Kadereit 2014; Kadereit et al. 2010; Sage 2004). In this family, C_3_–C_4_ intermediate species have been recorded in the genera *Salsola, Rhaphidophyton*, and *Sedobassia* (Freitag and Kadereit 2014; Schüssler et al. 2017; Voznesenskaya et al. 2001a, 2013; Wen and Zhang 2015).

The genus *Chenopodium* (Chenopodieae) is considered to include only C_3_ species (Jacobs 2001; Kadereit et al. 2010). This genus is cosmopolitan and includes about 150 species, most of which are annual herbs growing in arid and semi-arid regions and also on salt-rich soils (Fuentes-Bazan et al. 2012) and weeds of disturbed habitats and cultivated fields (Judd and Ferguson 1999). The seeds of some *Chenopodium* species such as *C. quinoa, C. berlandieri*, and *C. formosanum* are used as cereals, and the leaves and young shoots of *C. album* are eaten as vegetables (Judd and Ferguson 1999). *Chenopodium album* has been often used as a model plant to study the physiology of C_3_ photosynthesis (e.g., Haraguchi et al. 2009).

In our preliminary study on leaf anatomy of eudicot species in Japan, we have recently found that *C. album* has leaf structural traits of the proto-Kranz type, which clearly differ from those of the typical non-Kranz (C_3_) type. This finding motivated us to re-examine leaf anatomy and photosynthetic traits of *Chenopodium* species from various regions of the world. This study reports, for the first time, that *Chenopodium* includes proto-Kranz and C_3_–C_4_ intermediate types as well as C_3_ types and is therefore a valuable eudicot group to study the evolutionary and genetic transition from C_3_ to proto-Kranz to C_3_–C_4_ intermediate plants.

## Materials and methods

### Plant materials and growth conditions

The species and accessions of *Chenopodium* examined in this study are listed in Table 1. Seeds of two accessions of *C. album* were collected in upland fields of the National Institute of Agro-Environmental Sciences, Tsukuba, Ibaraki, Japan and along the roadside of Fukuoka City, Fukuoka, Japan. Seeds of *C. ficifolium* were also collected in upland fields of the National Institute of Agro-Environmental Sciences. Seeds of *C. quinoa* were provided by the NARO Genebank, Tsukuba, Japan. Seeds of five other accessions of *C. album* and 12 other species of *Chenopodium* were provided by the USDA Germplasm Resources, USA (Table 1). The seeds of C_4_ species of *Amaranthus* (*A. cruentus, A. dubius*, and *A. hybridus*), which were used as controls, were also a gift from the USDA Germplasm Resources (Table 1). All seeds were germinated in perforated multiwell nursery boxes filled with loam soil granules. Seedlings were grown for 3 weeks in a greenhouse at the experimental field of Kyushu University in July. The seedlings were then transplanted to 5-L pots (one plant per pot) with sandy loam soil containing nitrogen (ammonium nitrate), phosphorus (calcium superphosphate), and potassium (potassium chloride) fertilizers (1.0 g each). Plants were grown in a greenhouse [natural sunlight, wherein photosynthetic photon flux density (PPFD) at midday exceeded 1500 μmol m^−2^ s^−1^; 30–34 °C during the day and 24–27 °C during the night] for 1.5–2 months. Plants were watered daily. Fully expanded upper mature leaves taken from 3 plants per species (per accession for *C. album*) were used for analysis.

**Table 1:** List of *Chenopodium* species examined and control C_4_ species of *Amaranthus*

Species	Locality	Germplasm source	Accession No.
*Chenopodium album* L.	Arizona, USA	USDA Germplasm Resources	PI 666270
	Finland	USDA Germplasm Resources	PI 658748
	France	USDA Germplasm Resources	PI 262168
	Fukuoka, Japan	In the field	
	India	USDA Germplasm Resources	PI 658735
	Poznan, Poland	USDA Germplasm Resources	PI 658746
	Tsukuba, Japan	In the field	
*C. atrovirens* Rybd.	Arizona, USA	USDA Germplasm Resources	PI 666273
*C. berlandieri* ssp. *nuttalliae* (Staff.) H. D.	Puebla, Mexico	USDA Germplasm Resources	PI 433231
Wilson and Heiser			
*C. ficifolium* Sm.	Tsukuba, Japan	In the field	
*C. formosanum* Koidz.	Taiwan	USDA Germplasm Resources	PI 433378
*C. giganteum* D. Don	Oklahoma, USA	USDA Germplasm Resources	PI 596371
*C. hians* Standl.	New Mexico, USA	USDA Germplasm Resources	PI 666310
*C. incanum* (S. Watson) A. Heller	New Mexico, USA	USDA Germplasm Resources	PI 666313
*C. leptophyllum* (Moq.) Nutt. Ex S. Watson	Nevada, USA	USDA Germplasm Resources	Ames 29780
*C. nevadense* Standl.	Nevada, USA	USDA Germplasm Resources	PI 666321
*C. pallidicaule* Aellen	La Paz, Bolivia	USDA Germplasm Resources	PI 478406
*C. quinoa* Willd.	Tsukuba, Japan	NARO Genebank	JP No. 53591
*C. standleyanum* Aellen	Iowa, USA	USDA Germplasm Resources	PI 666323
*C. strictum* Roth	Saxony, Germany	USDA Germplasm Resources	PI 665284
*C. vulvaria* L	Santarem, Portugal	USDA Germplasm Resources	PI 614896
*Amaranthus cruentus* L.	California, USA	USDA Germplasm Resources	PI 647848
*A. dubius* Mart. Ex Thell.	Nepal	USDA Germplasm Resources	PI 619238
*A. hybridus* L.	Pennsylvania, USA	USDA Germplasm Resources	Ames 5580

### Anatomical and ultrastructural studies

Samples taken from the midsections of leaves (one leaf per plant) were fixed and embedded in Quetol resin (Nisshin EM, Shinjuku, Tokyo, Japan) as reported previously (Tsutsumi et al. 2017). Semithin sections (1 µm thickness) were cut with glass knives on an ultramicrotome (Reichert Ultracut S, Leica, Wien, Austria), mounted on glass slides, stained with 1% toluidine blue O, and observed under a light microscope (Eclipse Ci-L, Nikon Instech Co. Ltd., Tokyo, Japan). The profile areas of M and BS tissues between adjacent small vascular bundles were measured using the Image J software (National Institutes of Health, Bethesda, MD, USA), and the area ratio of M and BS tissues (M/BS tissue area ratio) was calculated. Simultaneously, the sizes (profile areas) of 8 M cells and 8 BS cells per plant were measured, and the size ratio of M and BS cells (M/BS cell size ratio) was calculated.

Ultrathin sections were cut with a diamond knife on the same ultramicrotome, picked up on Formvar-coated grids, stained with lead citrate, and viewed under a transmission electron microscope (JEM-100CX II K, JEOL Ltd., Tokyo, Japan) at 75 kV. The numbers and intracellular positions of chloroplasts and mitochondria were recorded for 5 M cells and 5 BS cells per plant as described by Hatakeyama and Ueno (2016). In BS cells, we counted chloroplasts and mitochondria in the inner halves of the cells (i.e., along the inner tangential wall and the inner half of the radial wall) and in the outer halves (i.e., along the outer tangential wall and the outer half of the radial wall). In M cells, we counted mitochondria on the vacuolar side of chloroplasts (inner position) and on the cell-wall side of chloroplasts, including isolated mitochondria not associated with chloroplasts but adjacent to the cell wall (outer position), as described in Hatakeyama and Ueno (2016). In M cells, chloroplasts in the intracellular position were not counted because all chloroplasts were adjacent to the cell wall. The numbers of chloroplasts and mitochondria per unit area were calculated in each of the 5 M and 5 BS cells using Image J software. On some sections, the sizes (profile areas) of 10 chloroplasts and 10 mitochondria per plant were measured.

To measure vein density, samples taken from the midsections of leaves (one leaf per plant) were fixed in a formalin–acetic acid–alcohol mixture and cleared in 80% lactic acid and chloral hydrate-saturated ethanol as described by Tsutsumi et al. (2017). The vein density (vein length per unit leaf area) was measured using Image J software.

### Immunohistochemistry

Intercellular immunolocalization of photorespiratory and photosynthetic enzymes in M and BS cells was investigated under a light microscope. Small leaf segments (one leaf per plant) were fixed and embedded in paraffin, as described by Hatakeyama and Ueno (2016). Sections (10 µm thick) were cut on a rotary microtome (PR-50, Yamato Kohki Industrial Co. Ltd., Saitama, Japan), mounted on slides coated with poly-l-lysine (Sigma-Aldrich Inc., St Louis, MO, USA), and dried overnight. Immunostaining for the P-protein of GDC (GDC-P) and the large subunit of Rubisco (Rubisco LSU) was performed as described by Hatakeyama and Ueno (2016) with antisera against GDC-P and Rubisco LSU from pea leaves. The antisera were provided by Dr. D. J. Oliver (University of Idaho, Moscow, ID, USA) and the late Dr. S. Muto (Nagoya University, Nagoya, Japan), respectively.

### Protein A—immunogold electron microscopy

To evaluate exactly the accumulation level of GDC-P in mitochondria of M and BS cells, a quantitative immunogold labeling study was made under an electron microscope. Small leaf segments (one leaf per plant) were fixed and embedded in Lowicryl K4 M resin (Chemische Werke Lowi GmbH, Waldkraiburg, Germany) as described by Ueno (1992). Ultrathin sections on Formvar-coated grids were immunolabeled with the antiserum against GDC-P and InnovaCoat Gold − 20 nm protein A nanoparticle conjugate (Innova Biosciences, Cambridge, England, UK), stained with lead citrate, and viewed under a transmission electron microscope as described by Ueno (1992). As a negative control, the antiserum was replaced by non-immune serum.

The density of GDC-P labeling was determined for mitochondria and other intracellular locations by counting the gold particles on electron micrographs at 20,000× magnification and calculating the number of particles per unit area (μm^−2^) with Image J software. We examined 13–18 mitochondria of palisade M cells and 20 mitochondria of BS cells in several sections per leaf. The density of labeling was calculated as the mean of 3 plants.

### Western blots

Leaves were frozen in liquid nitrogen and stored in a deep freezer (− 80 °C). Extraction of soluble proteins, SDS-PAGE, and Western blotting were performed as described by Ueno (1992) with antisera against phosphoenolpyruvate carboxylase (PEPC) and pyruvate, Pi dikinase (PPDK) from maize leaves (provided by Dr. T. Sugiyama, RIKEN, Yokohama, Japan). For GDC-P and Rubisco LSU, we used the same antisera for immunohistochemistry.

### Enzyme assays

Parts of frozen leaves were used to measure the activities of Rubisco, PEPC, NADP-malic enzyme (NADP-ME), and NAD-malic enzyme (NAD-ME) as described by Ueno (1992), except that all enzymes were assayed at 30 °C.

### Gas exchange measurements

Net CO_2_ assimilation rate (*A*) was measured using an LI-6400 portable photosynthesis system (Li-Cor Inc., Lincoln, NE, USA) at a PPFD of 1000 μmol m^−2^ s^−1^, a leaf temperature of 30 °C, a relative humidity of 60%, and a CO_2_ concentration of 380 μL L^−1^, as described in Ueno et al. (2003). Light within the chamber was provided by a 6400-02 LED Light Source (Li-Cor Inc.). The *Γ* value was determined by extrapolating the initial slope of *A* versus the intercellular CO_2_ concentration through the *x*-axis, where A equals zero.

### Carbon isotope ratio

One leaf from three plants was air-dried at 80 °C and ground in a mortar with a pestle. Two mg of leaf powder was used to measure ^12^C and ^13^C contents. Carbon isotope ratios were measured at SI Science, Kita-katsushika, Saitama, Japan by using the elemental analyzer**–**isotope ratio mass spectrometer (EA-IRMS) system (Thermo Fisher Scientific, Waltham, MA), as described by Sato and Suzuki (2010). The isotope ratio was expressed in δ notation as parts per million (‰) with respect to the Pee Dee belemnite standard.

### Statistical analysis

Data were presented as mean ± SD (*n* = 3 plants), except carbon isotope ratios. These data were analyzed using Statcel4 software (OMS Publisher, Tokorozawa, Saitama, Japan). We tested the significance (*P* < 0.05) of the differences in GDC-P labeling density between M and BS mitochondria using Student’s *t* test and that of the differences in structural, biochemical, and physiological traits among species and among photosynthetic types of *Chenopodium* by the Tukey–Kramer test as a post hoc test, associated with ANOVA. For enzyme activities, data of *Amaranthus* C_4_ species were added to statistical analysis. Pearson’s correlation coefficients between *Γ* values and quantitative parameters of cells and organelles were calculated.

## Results

### Leaf anatomy

**Fig. 1:**
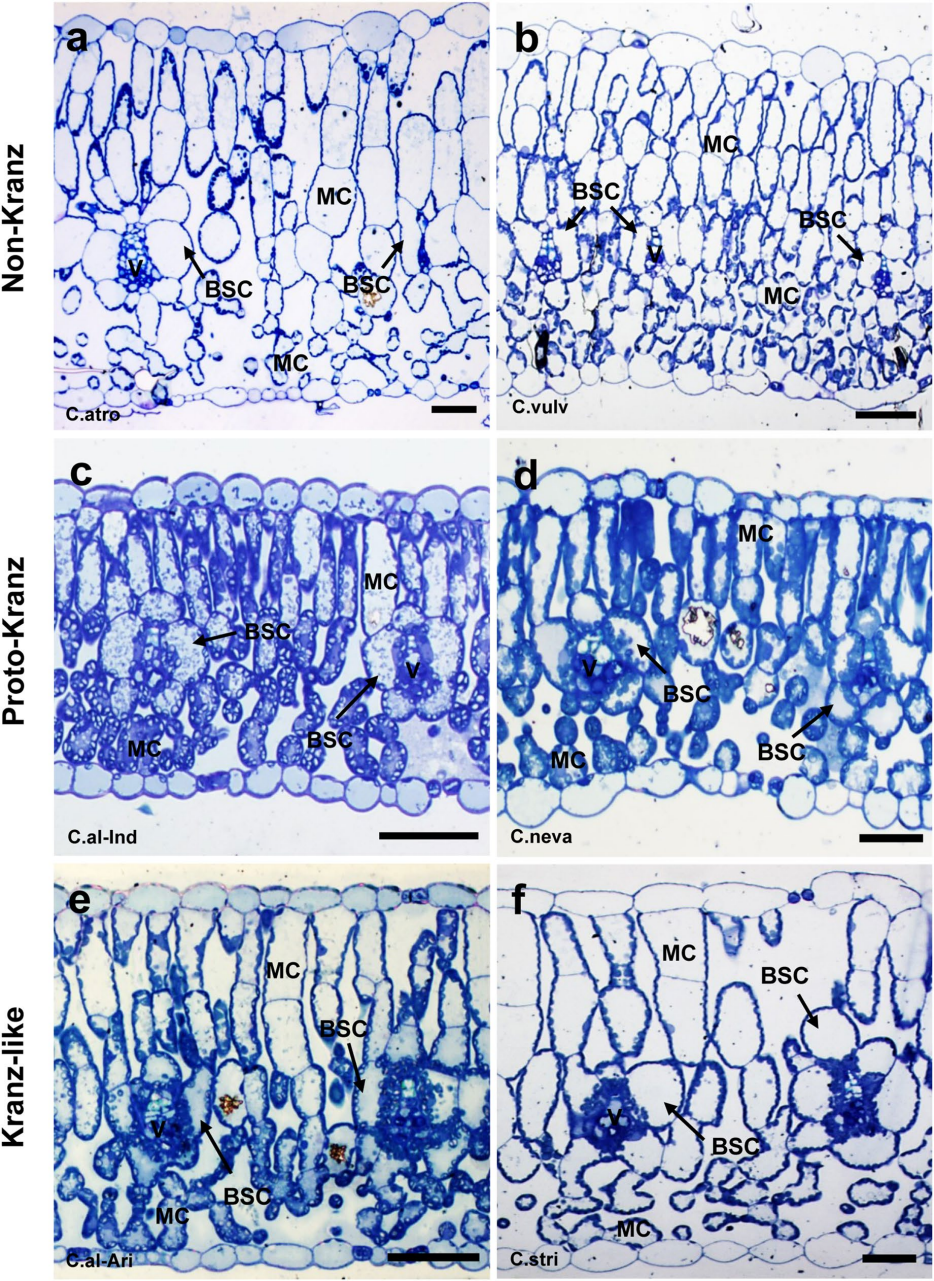
Leaf anatomy of *Chenopodium* species. **a***C. atrovirens*; **b***C. vulvaria*; **c***C. album* (India); **d***C. nevadense*; **e***C. album* (Arizona, USA); **f***C. strictum*. *BSC* bundle-sheath cell, *MC* mesophyll cell, *V* vascular bundle. Bars = 50 µm

Light microscopy revealed a large variation in chloroplast numbers and arrangement in BS cells among *Chenopodium* species examined (Fig. 1, S1–S3). We classified leaf anatomy of *Chenopodium* into 3 types: non-Kranz, proto-Kranz, and Kranz-like types (Fig. 1; Table 2). In non-Kranz anatomy (Fig. 1a, b, S1), BS cells contained few chloroplasts in the inner half (centripetal region) along the vascular bundle, but many chloroplasts occurred in the outer half adjacent to intercellular spaces. *Chenopodium atrovirens, C. hians, C. incanum, C. leptophyllum, C. pallidicaule, C. quinoa, C. standleyanum*, and *C. vulvaria* showed non-Kranz anatomy (Table 2). In proto-Kranz anatomy (Fig. 1c, d, S2, S3a–c), BS cells contained more chloroplasts in the centripetal region than did non-Kranz-type BS cells, and those chloroplasts surrounded the vascular bundle. Six accessions of *C. album, C. berlandieri, C. ficifolium, C. formosanum, C. giganteum*, and *C. nevadense* showed proto-Kranz anatomy (Table 2). In Kranz-like anatomy (Fig. 1e, f), BS cells contained many more chloroplasts in the centripetal region than did proto-Kranz-type BS cells, but fewer than did BS cells of a C_4_ species of *Amaranthus* (Fig. S3d). *Chenopodium strictum* and a *C. album* accession from Arizona showed Kranz-like anatomy (Table 2). In all 3 anatomical types, M was differentiated into palisade tissue on the adaxial side and spongy tissue on the abaxial side (Fig. 1, S1–S3).

**Table 2:** Leaf anatomy, immunolocalization of the P protein of glycine decarboxylase (GDC-P), carbon isotope ratios, net CO_2_ assimilation rate (*A*), and CO_2_ compensation point (*Γ*) in *Chenopodium* species and control C_4_ species of *Amaranthus*

Species (locality)	Leaf anatomy	GDC-P localization		δ^13^C	*A*	*Γ*
		M cells	BS cells	(‰)	(μmol m^−2^ s^−1^)	(μmol mol^−1^)
*C. atrovirens*	Non-Kranz	+++	−	− 32.6	19.8 ± 1.5^a^	65.5 ± 3.1^h^
*C. hians*	Non-Kranz	+++	−	− 31.3	22.0 ± 1.7^a^	49.0 ± 2.7^efg^
*C. incanum*	Non-Kranz	+	−	− 31.1	24.6 ± 4.6^a^	45.2 ± 3.0^cdef^
*C. leptophyllum*	Non-Kranz	+++	−	ND	ND	ND
*C. pallidicaule*	Non-Kranz	+++	+	− 29.4	15.3 ± 1.9^a^	56.5 ± 3.7^g^
*C. quinoa*	Non-Kranz	+++	+	ND	ND	ND
*C. standleyanum*	Non-Kranz	+++	+	− 27.5	19.9 ± 0.4^a^	49.9 ± 1.2^efg^
*C. vulvaria*	Non-Kranz	+++	−	− 29.3	20.5 ± 1.5^a^	55.6 ± 2.9^g^
Non-Kranz type average				− 30.2 ± 1.8*x*	20.4 ± 3.1*x*	53.6 ± 7.2*z*
*C. album* (Finland)	Proto-Kranz	++	+++	− 30.1	ND	ND
*C. album* (France)	Proto-Kranz	++	+++	− 30.6	15.0 ± 6.9^a^	48.3 ± 1.6^defg^
*C. album* (Fukuoka, Japan)	Proto-Kranz	+++	+++	ND	10.1 ± 3.0^a^	39.7 ± 1.4^bcd^
*C. album* (India)	Proto-Kranz	++	+++	− 29.0	18.3 ± 0.9^a^	38.1 ± 3.6^bc^
*C. album* (Poland)	Proto-Kranz	++	+++	− 29.2	23.9 ± 2.6^a^	42.9 ± 1.9^bcdef^
*C. album* (Tsukuba, Japan)	Proto-Kranz	+++	++	ND	12.7 ± 1.5^a^	38.1 ± 2.6^bc^
*C. berlandieri*	Proto-Kranz	+++	++	− 28.4	16.5 ± 2.0^a^	51.0 ± 3.2^fg^
*C. ficifolium*	Proto-Kranz	+++	++	ND	11.7 ± 2.0^a^	35.0 ± 0.9^b^
*C. formosanum*	Proto-Kranz	+++	++	− 27.9	18.6 ± 1.9^a^	42.5 ± 1.6^bcdef^
*C. giganteum*	Proto-Kranz	++	+++	− 26.7	16.0 ± 4.2^a^	41.7 ± 0.6^bcde^
*C. nevadense*	Proto-Kranz	++	+++	− 27.7	15.5 ± 1.1^a^	35.9 ± 1.0^b^
Proto-Kranz type average				− 28.7 ± 1.3*x*	15.8 ± 3.9*x*	41.3 ± 5.2*y*
*C. album* (Arizona, USA)	Kranz-like	−	+++	− 30.5	16.3 ± 0.7^a^	20.3 ± 6.2^a^
*C. strictum*	Kranz-like	−	+++	− 29.9	21.1 ± 5.3^a^	25.5 ± 4.1^a^
Kranz-like type average				− 30.2 ± 0.4*x*	18.7 ± 3.4*x*	22.9 ± 3.7*x*
*A. cruentus*	Kranz	−	+++	ND	ND	ND
*A. dubius*	Kranz	ND	ND	− 13.1	17.6 ± 4.9	Near 0

GDC-P localization: (+) and (−) refer to the relative intensities of staining, with (+++) indicating heavy staining and (−) indicating little or no staining. The values of *A* and *Γ* for each species/accession are given as the mean ± SD of three plants. Different letters indicate significant difference at *P *< 0.05

*M* mesophyll, *BS* bundle sheath, *ND* not determined

### Immunohistochemical localization of GDC-P and Rubisco LSU

**Fig. 2:**
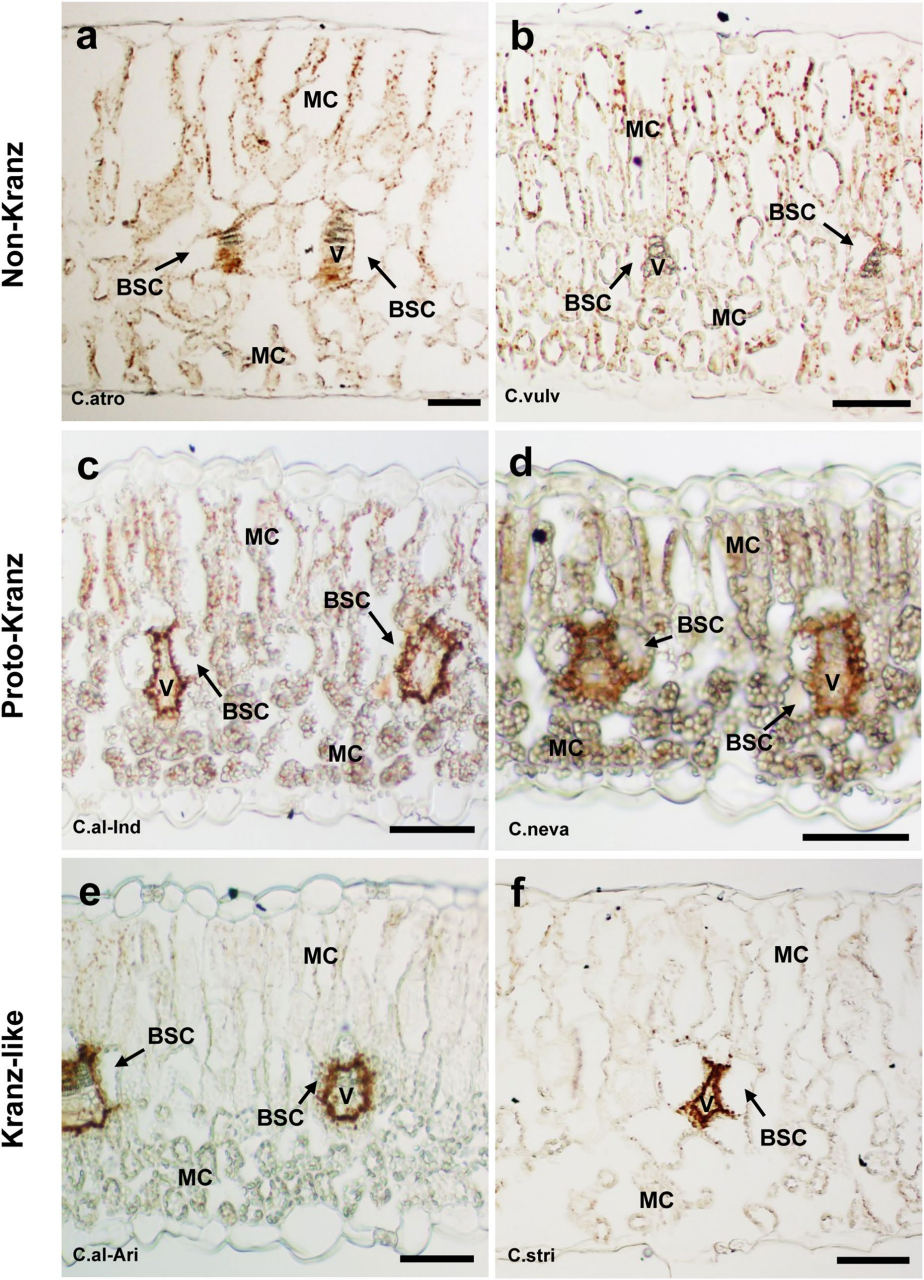
Immunohistochemical staining of GDC-P in mesophyll and bundle sheath cells of *Chenopodium* species. **a***C. atrovirens*; **b***C. vulvaria*; **c***C. album* (India); **d***C. nevadense*; **e***C. album* (Arizona, USA); **f***C. strictum*. *BSC* bundle-sheath cell, *MC* mesophyll cell, *V* vascular bundle. Bars = 50 µm

In the non-Kranz type, GDC-P was detected in M cells and to a lesser extent in BS cells (Fig. 2a, b, S4; Table 2). In the proto-Kranz type, GDC-P was detected in both M and BS cells (Fig. 2c, d, S5, S6a–c), but the degree of staining varied among species and *C. album* accessions (Table 2). In *C. giganteum*, *C. nevadense*, and four accessions of *C. album* (from Finland, France, India and Poland), the staining was stronger in BS cells than in M cells, and a distinct brown ring surrounded the vascular bundle; this ring represented a dense accumulation of GDC-P in mitochondria, as shown later by ultrastructural observation. In the *C. album* accession from Arizona and *C. strictum*, which had Kranz-like anatomy, GDC-P staining was detected exclusively in BS cells (Fig. 2e, f; Table 2). In a C_4_ species of *Amaranthus* also, GDC-P staining occurred exclusively in BS cells (Fig. S6d), as known in many C_4_ species (Yoshimura et al. 2004). In *Chenopodium* species, regardless of the anatomical type, Rubisco LSU was detected in chloroplasts of both M and BS cells (Fig. S7).

### CO_2_ gas exchange and carbon isotope ratio

As expected from leaf anatomy and GDC-P localization, *Chenopodium* species showed a large variation in *Г* (20–66 µL L^−1^; Table 2). These *Г* values were higher than that in a control C_4_ species, *Amaranthus dubius* (Table 2). In the Arizona accession of *C. album* and *C. strictum*, which have Kranz-like anatomy, the *Г* values were 20 and 26 µL L^−1^, respectively, which are typical for C_3_–C_4_ intermediates (Edwards and Ku 1987; Monson and Rawsthorne 2000). The *Г* values of non-Kranz species ranged from 45 to 66 µL L^−1^, whereas those of proto-Kranz species ranged from 35 to 51 µL L^−1^ (Table 2). The average *Г* values of the 3 anatomical types in *Chenopodium* significantly differed from each other. Relative to the average *Г* value of the non-Kranz type, the average *Г* value of the proto-Kranz type was 23% lower, and that of the Kranz-like type was 57% lower. The *A* values of *Chenopodium* species ranged from 10 to 25 µmol m^−2^ s^−1^, and that of *A. dubius* was 17.6 µmol m^−2^ s^−1^ (Table 2). The δ^13^C values of *Chenopodium* species ranged from − 32.6‰ to − 26.7‰, whereas that of *A. dubius* was − 13.1‰ (Table 2). These values were within C_3_ and C_4_ range, respectively (Ehleringer and Osmond 1991). The average *A* and δ^13^C values did not differ significantly among the anatomical types of *Chenopodium*.

### Activities and amounts of photosynthetic and photorespiratory enzymes

Activities of C_3_ and C_4_ photosynthetic enzymes were measured for five non-Kranz species, six proto-Kranz species (including 4 *C. album* accessions), the Arizona accession of *C. album* and *C. strictum* (both Kranz-like type), and two control C_4_ species of *Amaranthus* (Table 3). The average activities of Rubisco were higher in all anatomical types of *Chenopodium* than in the C_4_ species, whereas there were no significant differences among the three anatomical types of *Chenopodium*. In contrast, activities of PEPC were much lower in *Chenopodium* species than in the C_4_ species; there were no significant differences among the three anatomical types. Similar trends were found for NADP-ME and NAD-ME (Table 3). In several species, there were large differences in the enzyme activities among three plants examined, resulting in large standard deviation. It was considered that these differences were probably caused by those in growth rate of plants.

**Table 3:** Activities of photosynthetic enzymes in leaves of *Chenopodium* species and control C_4_ species of *Amaranthus*

Species (locality)	Rubisco (μmol mg chl^−1^ h^−1^)	PEPC (μmol mg chl^−1^ h^−1^)		NADP-ME (μmol mg chl^−1^ h^−1^)	NAD-ME (μmol mg chl^−1^ h^−1^)
*C. atrovirens*	479.1 ± 105.7^bedef^	27.9 ± 24.0^a^		4.0 ± 0.7^a^	4.1 ± 0.9^a^
*C. pallidicaule*	353.6 ± 115.3^abcd^	73.3 ± 4.0^a^		5.4 ± 2.3^ab^	19.8 ± 9.8^a^
*C. quinoa*	426.7 ± 60.2^abcdef^	124.5 ± 11.9^a^		ND	ND
*C. standleyanum*	308.5 ± 16.6^abe^	23.9 ± 1.2^a^		4.3 ± 2.1^a^	5.8 ± 1.4^a^
*C. vulvaria*	575.8 ± 120.4^cdef^	39.2 ± 33.5^a^		14.7 ± 3.5^ab^	31.7 ± 11.5^a^
Non-Kranz type average	428.7 ± 105.2*y*	57.8 ± 42.1*x*		7.1 ± 5.1*x*	15.4 ± 13.0*x*
*C. album* (India)	395.8 ± 151.3^abcdef^	34.3±2.0^a^		10.3 ± 6.3^ab^	11.3 ± 5.0^a^
*C. album* (Fukuoka, Japan)	239.3 ± 22.8^ab^	87.2 ± 7.3^a^		ND	ND
*C. album* (Poland)	645.0 ± 58.2^ef^	80.3 ± 10.5^a^		13.2 ± 7.5^ab^	10.8 ± 5.3^a^
*C. album* (Tsukuba, Japan)	411.0 ± 58.9^abcdef^	74.2 ± 22.2^a^		ND	ND
*C. berlandieri*	367.1 ± 24.6^abcd^	37.9 ± 7.0^a^		6.8 ± 0.7^ab^	22.5 ± 2.2^a^
*C. ficifolium*	474.4 ± 26.9^bcdef^	82.3 ± 6.9^a^		ND	ND
*C. formosanum*	377.8 ± 86.1^abcde^	33.2 ± 3.2^a^		6.6 ± 1.1^ab^	9.6 ± 1.6^a^
*C. giganteum*	178.8 ± 65.4^a^	33.7 ± 24.7^a^		3.7 ± 1.1^a^	6.8 ± 2.5^a^
*C. nevadense*	585.3 ± 82.1^def^	41.1 ± 9.7^a^		11.7 ± 3.4^ab^	25.3 ± 11.4^a^
Proto-Kranz type average	408.3 ± 148.2*y*	56.0 ± 24.0*x*		8.7 ± 3.6*x*	14.4 ± 7.6*x*
*C. album* (Arizona, USA)	382.4 ± 86.7^abcdef^	37.4 ± 36.9^a^		7.1 ± 1.1^ab^	6.0 ± 2.9^a^
*C. strictum*	654.2 ± 157.9^f^	71.8 ± 6.1^a^		5.6 ± 3.3^ab^	5.9 ± 4.0^a^
Kranz-like type average	518.3 ± 192.2*y*	54.6 ± 24.3*x*		6.4 ± 1.1*x*	6.0 ± 0.1*x*
*A. dubius*	193.8 ± 60.1^a^	596.5 ± 105.1^b^		19.6 ± 1.4^b^	82.8 ± 19.7^ab^
*A. hybridus*	231.1 ± 87.9^a^b	564.4 ± 138.8^b^		45.0 ± 23.1^c^	201.3 ± 155.1^b^
C_4_ type average	212.5 ± 70.4*x*	580.5 ± 111.5*y*		32.3 ± 20.2*y*	142.0 ± 118.3*y*

For each species, values are given as the mean ± SD of three plants. Different letters indicate significant difference at *P* < 0.05

*ND* not determined

**Fig. 3:**
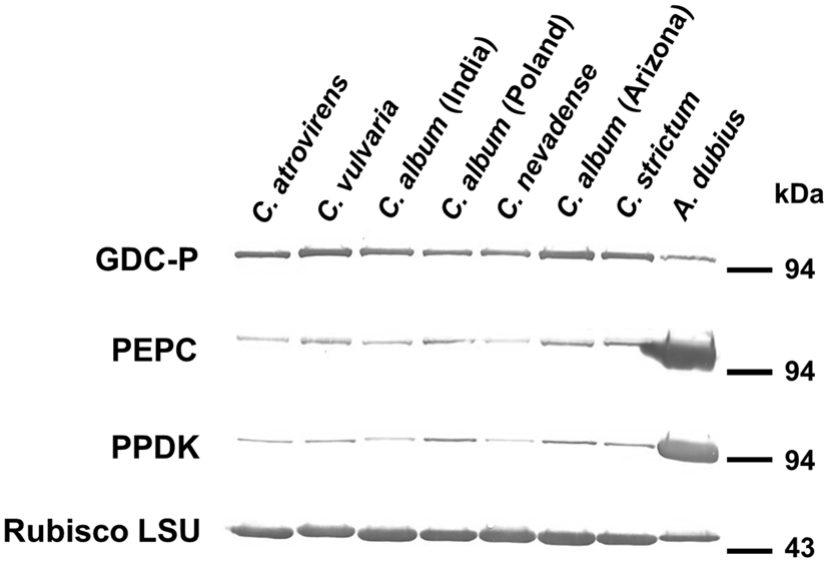
Western blots of leaf extracts of *Chenopodium* species. Total soluble protein (20 µg for GDC-P, PEPC, and PPDK and 2.5 µg for Rubisco LSU) was subjected to SDS-PAGE, blotting on nitrocellulose membranes, and identification with antisera against the indicated photorespiratory and photosynthetic enzymes

Western blot analyses of photosynthetic and photorespiratory enzymes were done for five species of *Chenopodium* (three accessions from *C. album*) representing the three anatomical types and a control C_4_ species, *A. dubius* (Fig. 3). The levels of Rubisco LSU and GDC-P were higher, and those of PEPC and PPDK were much lower, in *Chenopodium* than in *A. dubius*.

### Quantification of leaf inner structure

**Fig. 4:**
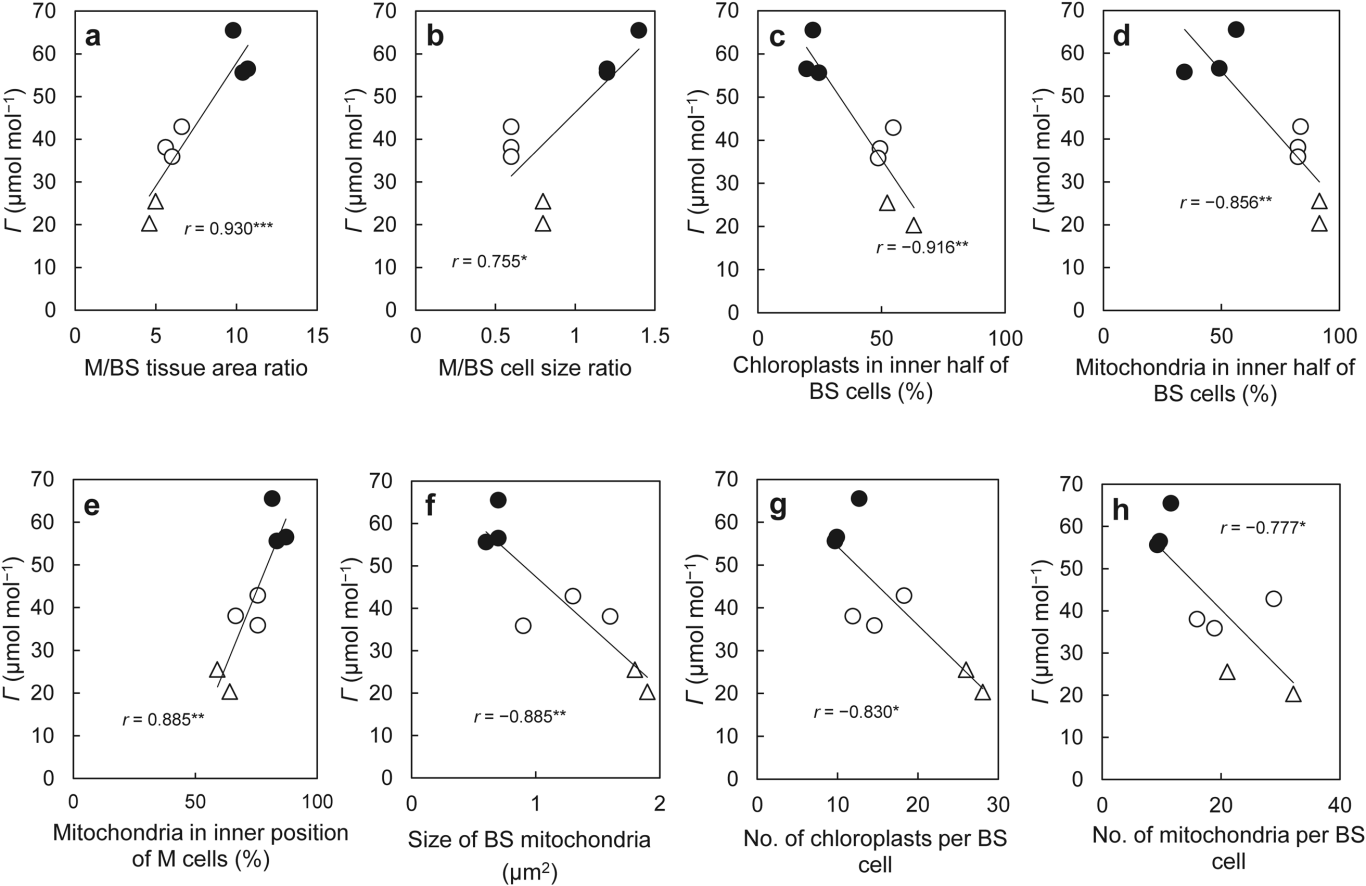
Relationships between CO_2_ compensation point (*Г*) and M/BS tissue area ratio (**a**), M/BS cell size ratio (**b**), the ratio of chloroplasts located in the inner half of BS cells (**c**), the ratio of mitochondria located in the inner half of BS cells (**d**), the ratio of mitochondria located in the inner position of M cells (**e**), size of BS mitochondria (**f**), number of chloroplasts per BS cell (**g**), and number of mitochondria per BS cell (**h**) in *Chenopodium* species. Filled circles, non-Kranz type; white circles, proto-Kranz type; triangles, Kranz-like type. Significant at *P:* * < 0.05; ** < 0.01; *** < 0.001

We investigated the leaf inner structure of the representatives of the three anatomical types in more detail (Table 4). There were no significant differences in vein density among the three anatomical types. The sectional area of M tissue was highest in the non-Kranz type and lowest in the Kranz-like type, whereas that of BS tissue showed a reverse tendency. As a result, the M/BS tissue area ratio was higher in the non-Kranz type than in the proto-Kranz and Kranz-like types (Table 4). The size of M and BS cells varied greatly among *Chenopodium* species. For example, the size of M cells in *C. atrovirens* was more than five times that in the Indian accession of *C. album*. Nevertheless, the M/BS cell size ratio was almost constant among species of the same anatomical type but was lowest in the proto-Kranz type and highest in the non-Kranz type (Table 4). There was a high positive correlation between *Г* and the M/BS tissue area ratio (Fig. 4a) and a weaker positive correlation between *Г* and the M/BS cell size ratio (Fig. 4b).

**Table 4:** Structural traits of leaves in *Chenopodium* species

Species (locality)	Vein density (mm mm^−2^)	M tissue area (%)	BS tissue area (%)	M/BS tissue area ratio	M cell size (μm^2^)	BS cell size (μm^2^)	M/BS cell size ratio
*C. atrovirens*	6.7 ± 2.0^ab^	90.4 ± 2.3^cd^	9.6 ± 2.3^ab^	9.8 ± 2.3^b^	1723.7 ± 507.5^b^	1287.9 ± 248.3^c^	1.4 ± 0.3^d^
*C. pallidicaule*	ND	91.5 ± 0.6^d^	8.5 ± 0.6^a^	10.7 ± 0.7^b^	710.5 ± 28.4^a^	579.0 ± 47.5^a^	1.2 ± 0.1^cd^
*C. vulvaria*	7.0 ± 2.0^ab^	91.2 ± 0.5^d^	8.8 ± 0.5^a^	10.4 ± 0.7^b^	732.5 ± 97.1^a^	615.7 ± 90.8^ab^	1.2 ± 0.2^bcd^
Non-Kranz type average	6.7 ± 0.2*x*	91.5 ± 0.3*x*	9.0 ± 0.5*x*	10.3 ± 0.5*y*	1055.6 ± 578.7*y*	827.5 ± 399.1*x*	1.3 ± 0.1*z*
*C. album* (India)	9.4 ± 2.1^c^	84.9 ± 1.1^ab^	15.1 ± 1.1^cd^	5.6 ± 0.5^a^	293.7 ± 66.7^a^	510.0 ± 75.9^a^	0.6 ± 0.1^a^
*C. album* (Poland)	6.7 ± 2.0^ab^	86.8 ± 1.6^bc^	13.2 ± 1.6^bc^	6.6 ± 0.9^a^	681.7 ± 88.5^a^	1225.4 ± 336.3^bc^	0.6± 0.1^a^
*C. nevadense*	8.5 ± 2.3^c^	85.7 ± 1.0^ab^	14.3 ± 1.0^cd^	6.0 ± 0.5^a^	386.9 ± 14.1^a^	784.1 ± 385.9^ab^	0.6 ± 0.1^a^
Proto-Kranz type average	8.2 ± 1.4*x*	85.8 ± 0.9*y*	14.2 ± 0.9^y^	6.1 ± 0.7*x*	454.1 ± 202.5*x*	839.8 ± 360.9*x*	0.6 ± 0.0*x*
*C. album* (Arizona)	11.8 ± 2.6^d^	82.2 ± 1.3^a^	17.8 ± 1.3^d^	4.6 ± 0.2^a^	636.4 ± 156.8^a^	800.5 ± 217.0^ab^	0.8 ± 0.0^abc^
*C. strictum*	5.8 ± 1.4^ab^	83.1 ± 1.7^ab^	16.9 ± 1.7^cd^	5.0 ± 0.4^a^	1450.2 ± 165.2^b^	1898.1 ± 359.9^c^	0.8 ± 0.1^ab^
Kranz-like type average	8.8 ± 4.2*x*	82.7 ± 0.7*z*	17.3 ± 0.7*z*	4.8 ± 0.5*x*	1043.3 ± 575.5*y*	1349.3 ± 776.1*x*	0.8 ± 0.0*y*

The values for each species/accession are given as the mean ± SD of three plants. Different letters indicate significant difference at *P* < 0.05

*M* mesophyll, *BS* bundle sheath

### Ultrastructure and quantification of organelles

**Fig. 5:**
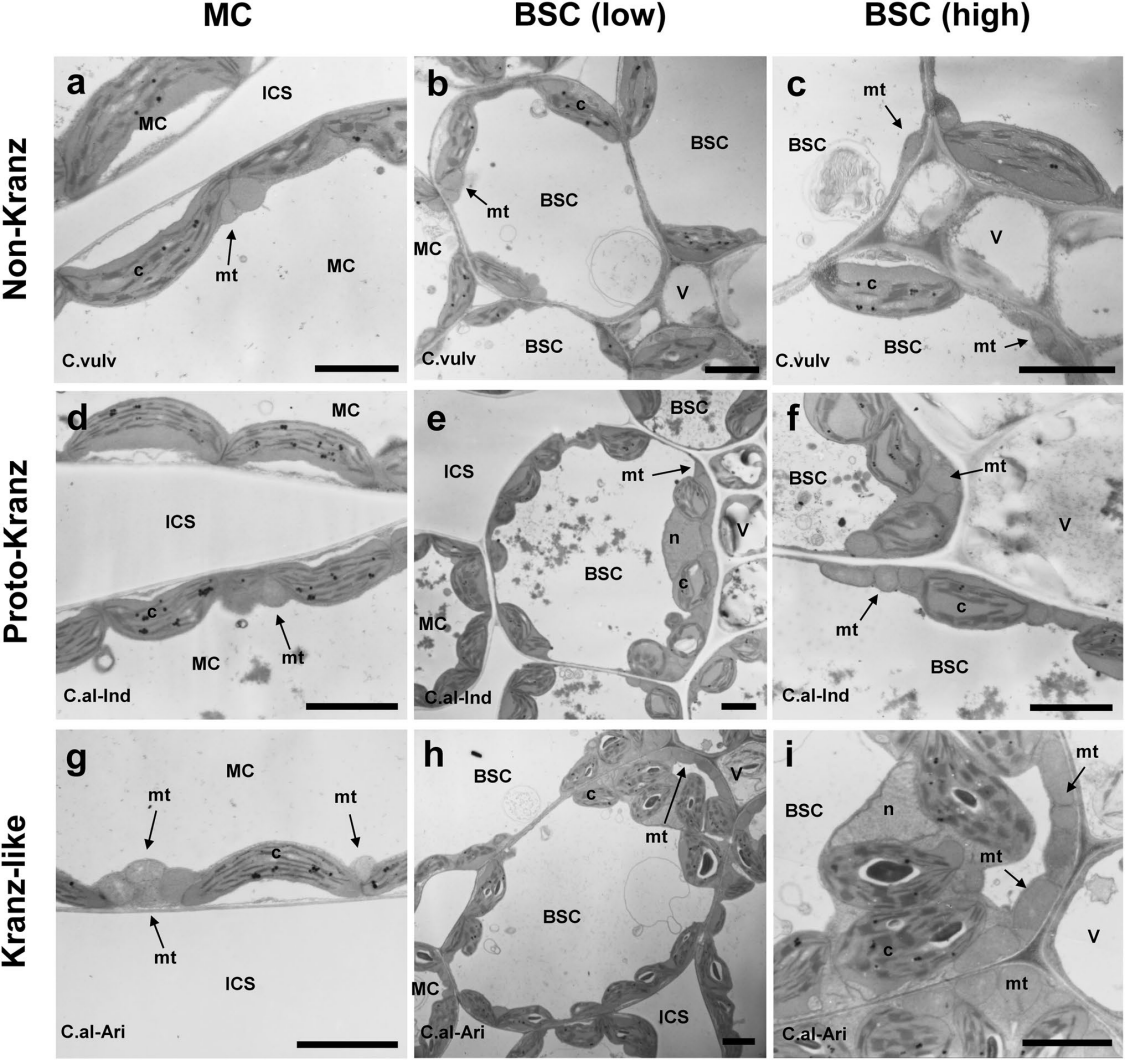
Ultrastructure of mesophyll cells (**a**, **d**, **g**) and bundle-sheath cells at a low (**b**, **e**, **f**) and at a high (**c**, **f**, **i**) magnification in *Chenopodium* species. (**a**–**c**) *C. vulvaria*; (**d**–**f**) *C. album* (India); (**g**–**i**) *C. album* (Arizona, USA). *BSC* bundle-sheath cell, *ICS* intercellular space, *MC* mesophyll cell, *V* vascular bundle, *c* chloroplast, *mt* mitochondrion, *n* nucleus. Bars = 3 µm

In the non-Kranz type, only a few chloroplasts and mitochondria were located in the centripetal region of BS cells (Fig. 5b, c, S8b, c, e, f). In the proto-Kranz type, more chloroplasts and mitochondria were located in the centripetal region, where many mitochondria were located between chloroplasts and vascular tissues (Fig. 5e, f, S8 h, i, k, l). In the Kranz-like type, the preferential localization of these organelles in the centripetal region was most pronounced (Fig. 5h, i, S8n, o). The ratio of chloroplasts and mitochondria in the inner half of BS cells was higher in both proto-Kranz and Kranz-like types than in the non-Kranz type (Table 5). In the M cells of the non-Kranz type, most mitochondria were located on the vacuolar side of chloroplasts (in the inner position) (Fig. 5a; Table 5; see Fig. 5d, g for the proto-Kranz and Kranz-like types). The ratio of mitochondria in the inner position decreased from the non-Kranz to the proto-Kranz to the Kranz-like type (Table 5). There were significant correlations between *Г* and the centripetal positioning of chloroplasts and mitochondria in BS cells (Fig. 4c, d) and between *Г* and the inner positioning of M mitochondria (Fig. 4e).

**Table 5:** Intracellular position of mitochondria and chloroplasts in mesophyll and bundle sheath cells of *Chenopodium* species

	M cells	BS cells	
Species (locality)	Mitochondria	Chloroplasts	Mitochondria
	Inner position (%)	Inner half of cell (%)	Inner half of cell (%)
*C. atrovirens*	81.6 ± 2.5^bc^	22.2 ± 7.9^a^	56.3 ± 5.7^b^
*C. pallidicaule*	87.2 ± 6.3^c^	19.7 ± 4.0^a^	49.1 ± 8.3^ab^
*C. vulvaria*	83.5 ± 2.5^bc^	24.7 ± 10.4^a^	34.4 ± 11.6^a^
Non-Kranz type average	84.1 ± 4.4*z*	22.2 ± 2.5*x*	46.6 ± 11.1*x*
*C. album* (India)	66.6 ± 7.9^ab^	49.3 ± 2.8^b^	82.2 ± 2.6^c^
*C. album* (Poland)	75.7 ± 9.8^abc^	54.7 ± 0.5^b^	83.4 ± 4.3^c^
*C. nevadense*	75.6 ± 2.6^abc^	48.6 ± 1.4^b^	82.3 ± 3.2^c^
Proto-Kranz type average	72.6 ± 7.9*y*	50.9 ± 3.3*y*	82.6 ± 3.0*y*
*C. album* (Arizona)	64.2 ± 8.6^a^	63.1 ± 11.0^b^	91.5 ± 4.2^c^
*C. strictum*	59.1 ± 3.0^a^	52.3 ± 6.9^b^	91.5 ± 4.3^c^
Kranz-like type average	61.6 ± 6.4*x*	57.9 ± 7.3*y*	91.5 ± 3.8*y*

The values for each species/accession are given as the mean ± SD of three plants. Different letters indicate significant difference at *P *< 0.05

*M* mesophyll, *BS* bundle sheath

In M cells, the size of chloroplasts and mitochondria and the number of chloroplasts and mitochondria per cell did not differ among the three types, and their numbers per unit area were higher in the proto-Kranz type than in the other two types (Table 6). In BS cells, the number of chloroplasts per cell was highest in the Kranz-like type, and the number of mitochondria was lowest in the non-Kranz type (Table 6). The numbers of these organelles per unit area tended to be lowest in the non-Kranz type (Table 6). In BS cells, the size of chloroplasts did not differ significantly among the three types, and the mitochondria were smallest in the non-Kranz and largest in the Kranz-like type (Table 6). There were significant negative correlations between *Г* and the size of BS mitochondria (Fig. 4f), the number of chloroplasts and mitochondria per BS cell (Fig. 4g, h).

**Table 6:** Quantitative traits of chloroplasts and mitochondria in mesophyll and bundle sheath cells of *Chenopodium* species

Organelles and species (locality)	M cells			BS cells		
	Size (μm^2^)	No. per cell	No. per cell area (μm^−2^×10^−3^)	Size (μm^2^)	No. per cell	No. per cell area (μm^−2^×10^−3^)
Chloroplasts						
*C. atrovirens*	9.4 ± 1.3^abc^	19.9 ± 3.6^b^	11.5 ± 2.1^a^	7.8 ± 2.1^a^	12.7 ± 1.1^ab^	9.8 ± 0.9^a^
*C. pallidicaule*	6.5 ± 0.9^a^	16.8 ± 0.6^ab^	23.6 ± 0.8^c^	6.7 ± 0.3^a^	9.9 ± 1.3^a^	17.0 ± 2.3^abc^
*C. vulvaria*	7.7 ± 0.8^ab^	16.8 ± 1.2^ab^	22.9 ± 1.6^bc^	7.5 ± 1.5^a^	9.7± 1.2^a^	15.7 ± 2.0^abc^
Non-Kranz type average	7.9 ± 1.5*x*	17.8 ± 1.8*x*	19.4 ± 6.8*x*	7.3 ± 0.6*x*	10.7 ±1.7*x*	14.2 ± 3.8*x*
*C. album* (India)	7.9 ± 1.0^ab^	15.9 ± 2.7^ab^	54.3 ± 9.3^e^	6.6 ± 0.7^a^	11.9 ± 1.9^ab^	23.3 ± 3.7^bc^
*C. album* (Poland)	9.8 ± 1.3^bc^	16.7 ± 1.1^ab^	24.5 ± 1.6^c^	9.0 ± 1.9^a^	18.3 ± 2.4^bc^	15.0 ± 2.0^abc^
*C. nevadense*	9.0 ± 1.3^abc^	14.7 ± 0.5^a^	38.1 ± 1.2^d^	9.8 ± 2.0^a^	14.6 ± 2.6^ab^	23.7 ± 4.1^c^
Proto-Kranz type average	8.9 ± 1.0*x*	15.8 ± 1.7*x*	39.0 ± 14.9*y*	8.5 ± 2.0*x*	14.9 ± 3.2*x*	20.6 ± 5.2*xy*
*C. album* (Arizona)	12.0 ± 0.8^c^	17.5 ± 0.8^ab^	27.4 ± 1.3^c^	7.3 ± 0.7^a^	28.1 ± 5.5^d^	35.1 ± 0.9^d^
*C. strictum*	8.6 ± 0.9^ab^	19.5 ± 0.5^ab^	13.4 ± 0.3^ab^	9.2 ± 0.9^a^	26.0 ± 3.7^cd^	13.7 ± 2.0^ab^
Kranz-like type average	10.3 ± 2.5*x*	18.5 ± 1.2*x*	20.4 ± 9.9*x*	8.2 ± 1.3*x*	27.1 ± 1.5*y*	24.4 ± 12.6*y*
Mitochondria						
*C. atrovirens*	1.0 ± 0.1^a^	13.9 ± 8.8^a^	8.1 ± 5.1^ab^	0.7 ± 0.2^a^	11.6 ± 2.2^a^	9.0 ± 1.7^a^
*C. pallidicaule*	0.6 ± 0.1^a^	12.8 ± 2.5^a^	18.0 ± 3.5^ab^	0.7 ± 0.0^a^	9.7 ± 1.4^a^	16.8 ± 2.4^ab^
*C. vulvaria*	0.7 ± 0.2^a^	7.8 ± 1.6^a^	10.6 ± 2.2^ab^	0.6 ± 0.1^a^	9.3 ± 3.0^a^	15.0 ± 4.9^a^
Non-Kranz type average	0.8 ± 0.2*x*	11.5 ± 3.3*x*	12.2 ± 5.2*x*	0.7 ± 0.1*x*	10.2 ± 2.3*x*	13.6 ± 4.1*x*
*C. album* (India)	0.7 ± 0.1^a^	7.5 ± 2.9^a^	25.4 ± 10.0^b^	1.6 ± 0.3^bc^	16.0 ± 3.5^ab^	31.4 ± 6.8^bc^
*C. album* (Poland)	0.8 ± 0.3^a^	17.1 ± 7.8^a^	25.0 ± 11.4^b^	1.3± 0.4^abc^	28.9 ± 9.2^bc^	23.6 ± 7.5^ab^
*C. nevadense*	0.7 ± 0.3^a^	10.7 ± 1.9^a^	27.6 ± 5.0^c^	0.9 ± 0.4^ab^	18.9 ± 3.2^abc^	30.7 ± 5.1^bc^
Proto-Kranz type average	0.7 ± 0.1*x*	11.7 ± 4.9*x*	26.0 ± 1.4*y*	1.3 ± 0.5*y*	21.3 ± 6.7*y*	28.5 ± 4.3*y*
*C. album* (Arizona)	1.0 ± 0.2^a^	7.5 ± 1.8^a^	11.8 ± 2.8^ab^	1.9 ± 0.2^c^	32.2 ± 6.4^c^	40.2 ± 8.0^c^
*C. strictum*	0.8 ±0.2^a^	8.3 ± 3.4^a^	5.7 ± 2.3^a^	1.8 ± 0.1^c^	21.1 ± 5.6^abc^	11.2 ± 3.0^a^
Kranz-like type average	0.9 ± 0.2*x*	7.9 ± 0.6*x*	8.8 ± 4.3*x*	1.8 ± 0.2*z*	26.7 ± 8.1*y*	25.7 ± 16.8*xy*

The values for each species/accession are given as the mean ± SD of three plants. Different letters indicate significant difference at *P* < 0.05

*M* mesophyll, *BS* bundle sheath

### Immunogold localization of GDC-P

**Fig. 6:**
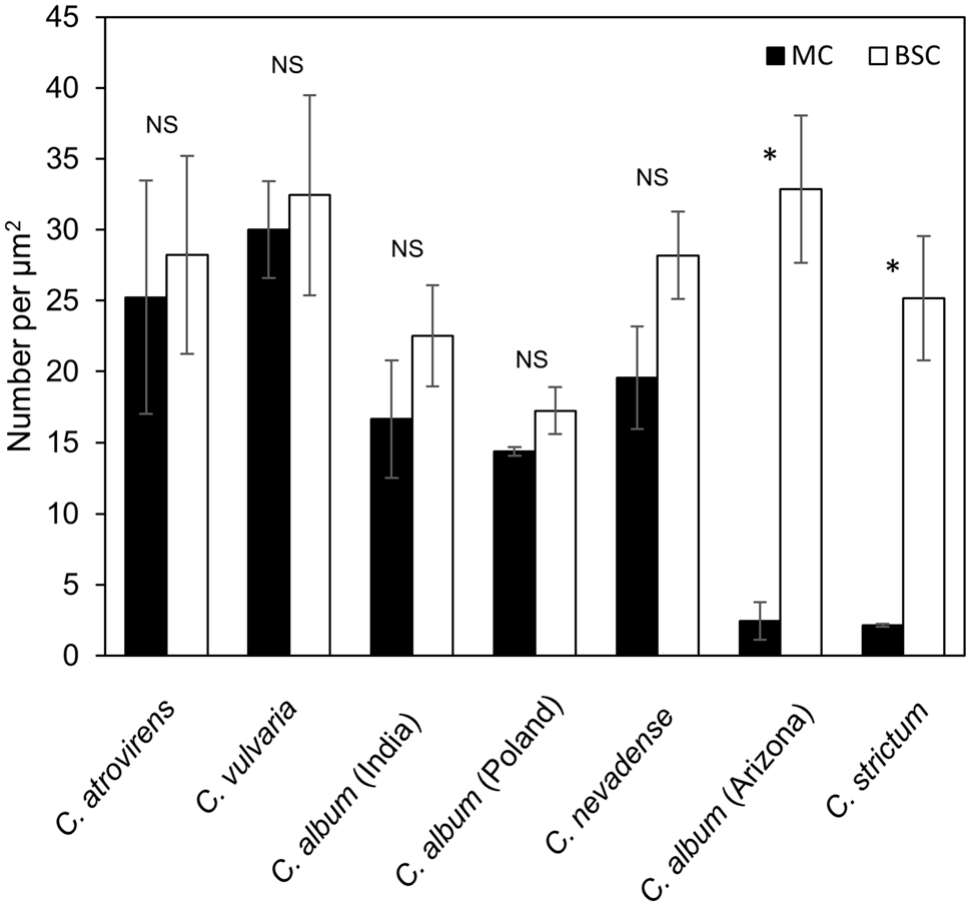
Quantification of immunogold labeling of GDC-P in the mitochondria of mesophyll cells (MC) and bundle-sheath cells (BSC) of *Chenopodium* species. Numbers of gold particles per unit area of mitochondria are given as mean ± SD of three plants. * significant differences between MC and BSC at *P* < 0.05. *NS* not significant. Labeling densities in the cell area excluding mitochondria were between 0.05 and 0.15 µm^−2^

In the non-Kranz and proto-Kranz types, GDC-P was detected in the mitochondria of both M and BS cells (Fig. S9a–d). In both types, the labeling density did not differ significantly between M and BS mitochondria (Fig. 6). In the Kranz-like type, GDC-P was detected almost exclusively in BS mitochondria (Fig. 6, S9e, f).

## Discussion

### Photosynthetic types in *Chenopodium*

Although *Chenopodium* is considered to be a C_3_ genus, our study revealed that *Chenopodium* species show great variation in leaf anatomy, and some species have biochemical and physiological traits characteristic of C_3_–C_4_ intermediates. Light microscopy showed a difference in chloroplast number in the centripetal region of BS cells, an indication of the existence of non-Kranz, proto-Kranz, and Kranz-like species in this genus (Table 2). Gas exchange measurements showed that the non-Kranz type had the highest *Г* values, typical of C_3_ plants, whereas the Kranz-like type had the lowest *Г* values, typical of C_3_–C_4_ intermediate plants (Edwards and Ku 1987). GDC-P was found mainly in M cells in the non-Kranz type and exclusively in BS cells in the Kranz-like type. The latter GDC-P distribution is responsible for the operation of the glycine shuttle (Monson and Rawsthorne 2000; Rawsthorne 1992; Rawsthorne et al. 1988). These data suggest that the Arizona accession of *C. album* and *C. strictum* (Kranz-like type) are C_3_–C_4_ intermediates.

We found numerous proto-Kranz species, intermediate between the non-Kranz and Kranz-like types (Table 2). The BS cells of proto-Kranz species also contained a considerable number of chloroplasts in the centripetal region, but the number was somewhat lower than in the BS cells of the Kranz-like type. In the proto-Kranz type, GDC-P immunostaining was detected in both M and BS cells, but the relative intensity of staining between these cells varied among species (Table 2). These *Chenopodium* species resemble the proto-Kranz plants previously found in some genera, such as *Heliotropium* (Muhaidat et al. 2011; Vogan et al. 2007), *Flaveria* (Sage et al. 2013), *Salsola* (Schüssler et al. 2017; Voznesenskaya et al. 2013), and *Steinchisma* (Brown et al. 1983; Khoshravesh et al. 2016).

In comparison with the C_4_*Amaranthus* species, *Chenopodium* species had higher activities of the C_3_ enzyme Rubisco and lower activities of the C_4_ enzymes PEPC, NADP-ME and NAD-ME (Table 3). Western blot analysis showed that *Chenopodium* species accumulated smaller amounts of PEPC and PPDK but greater amounts of Rubisco and GDC than did *Amaranthus* species. These data suggest that *Chenopodium* species perform photosynthesis without contribution of the C_4_ cycle. Rubisco accumulated in all chloroplasts of M and BS cells. We concluded that the Arizona accession of *C. album* and *C. strictum* (Kranz-like anatomy) are type I C_3_–C_4_ intermediates, *Chenopodium* species with non-Kranz anatomy are C_3_, and other species of *Chenopodium* and the remaining accessions of *C. album* are of the proto-Kranz type. This conclusion is also supported by δ^13^C values of *Chenopodium* species (Table 2). Previous studies reported that type I C_3_–C_4_ intermediates and proto-Kranz type have C_3_-like δ^13^C values (Edwards and Ku 1987; Vogan et al. 2007), as in *Chenopodium* species. This is due to these plants originally fixing CO_2_ via Rubisco, not via the C_4_ cycle (von Caemmerer and Hubick 1989).

The genus *Chenopodium* is polyphyletic and combines species from three clades of Chenopodioideae (Kadereit et al. 2003). Our study investigated 15 species, which is only about 10% of the known *Chenopodium* species. From these limited data, it would be difficult to reliably deduce on the phylogenetic relationships among photosynthetic types in *Chenopodium*, and a more extensive survey would be required. In general, most C_3_–C_4_ intermediate species have been found in genera that include C_4_ species (Sage et al. 2014), but as far as we know no C_4_ species has been identified in *Chenopodium*. A few genera include C_3_–C_4_ intermediate species with C_3_ species (e.g., *Moricandia, Diplotaxi*s, and *Brassica* in Brassicaceae, Schlüter et al. 2017; Ueno 2011; *Parthenium* in Compositae, Moore et al. 1987).

### Transition of leaf structural and photosynthetic traits in *Chenopodium*

Our study revealed a gradation of structural and photosynthetic traits from C_3_ to proto-Kranz to C_3_–C_4_ intermediate type in *Chenopodium*. Muhaidat et al. (2007) found no significant difference in vein density between closely related C_3_ and C_4_ species of eudicots. In *Chenopodium*, we also found no significant differences in vein density among the three types, but the great variation in size of M and BS cells among *Chenopodium* species might affect the tendency of changes in vein density (Table 4). The M/BS tissue area ratio is higher in C_3_ species than in C_4_ species (Hattersley 1984; Muhaidat et al. 2011). In *Chenopodium*, the M/BS tissue area ratio decreased from C_3_ (non-Kranz) to proto-Kranz to C_3_–C_4_ intermediate (Kranz-like) type (Table 4; Fig. 4a). The M/BS cell size ratio showed a similar trend (Table 4; Fig. 4b). Therefore, volume changes at the tissue and cell levels appear to occur during the transition from C_3_ to proto-Kranz to C_3_–C_4_ intermediate species in *Chenopodium*, although the differences between the proto-Kranz and C_3_–C_4_ intermediates were somewhat indistinct.

In the BS cells of C_3_–C_4_ intermediate plants, the amount and positioning of chloroplasts and mitochondria are critical structural traits involved in photosynthesis (Brown and Hattersley 1989; Edwards and Ku 1987; Muhaidat et al. 2011; Rawsthorne 1992; Sage et al. 2013; Ueno et al. 2003; Ueno 2011; Voznesenskaya et al. 2013). In the BS cells of *Chenopodium*, the size of mitochondria increased from C_3_ to proto-Kranz to C_3_–C_4_ intermediate species (Fig. 4f), but there was no significant difference in chloroplast size (Table 6). The numbers of chloroplasts and mitochondria per cell and per unit cell area tended to be lowest in C_3_ species (Table 6; Fig. 4g, h). The distribution of chloroplasts and mitochondria to the inner half of BS cells (centripetal positioning) was also lowest in C_3_ species (Table 5; Fig. 4c, d), as reported for other genera (Brown et al. 1983; Khoshravesh et al. 2016; Muhaidat et al. 2011; Rawsthorne 1992; Sage et al. 2013; Ueno 2011; Ueno et al. 2003; Voznesenskaya et al. 2013). The mitochondria in BS cells were located between the centripetally located chloroplasts and the inner tangential walls. These structural features would help to capture photorespiratory CO_2_ released from mitochondria and to suppress the escape of CO_2_ from BS cells (Rawsthorne 1992; Sage et al. 2014).

In M cells, the size of chloroplasts and mitochondria and number of chloroplasts and mitochondria per cell did not differ among the three types, but the degree of inner positioning of mitochondria gradually decreased from C_3_ to proto-Kranz to C_3_–C_4_ intermediate species (Table 6; Fig. 4e). In contrast, the immunogold labeling density of GDC did not differ significantly between the M and BS mitochondria of C_3_ and proto-Kranz species, but in C_3_–C_4_ intermediates, GDC accumulated exclusively in BS mitochondria (Fig. 6). Most mitochondria in M cells are located on the vacuolar side of chloroplasts (inner position) in C_3_ grasses (Busch et al. 2013; Hatakeyama and Ueno 2016; Sage and Sage 2009) but are adjacent to the cell wall (outer position) in C_4_ grasses (Hatakeyama and Ueno 2017). This difference is associated with the difference in localization of GDC and Rubisco (these enzymes are present in C_3_ M cells but absent in C_4_ M cells) and thereby the difference in the requirement for scavenging of photorespiratory CO_2_ released from mitochondria (Hatakeyama and Ueno 2017). This relationship between mitochondria positioning and photosynthetic types in grasses appears to be also applicable to *Chenopodium* species.

Our data suggest that the main structural and biochemical events during the transition from C_3_ to proto-Kranz to C_3_–C_4_ intermediate type in *Chenopodium* are (1) the increase in BS relative to M tissue area (volume); (2) the increase in the number of chloroplasts and mitochondria in BS cells; (3) the increase in the distribution of these organelles to the centripetal region of BS cells; (4) the enlargement of BS mitochondria; and (5) the increase in the level of GDC in BS relative to M tissue. These changes allow limited operation of the glycine shuttle in the proto-Kranz type and full operation of the shuttle in the C_3_–C_4_ intermediate type, resulting in decreasing *Г* values (Fig. 4). Our data on the proto-Kranz type also clearly indicate that a complete suppression of GDC-P expression in M cells is not required for the reduction in *Г*, consistent with our previous studies on artificial hybrids with different genome constitution between C_3_–C_4_ intermediate and C_3_ species of Brassicaceae (Ueno et al. 2003). The average *Г* value in the proto-Kranz type of *Chenopodium* was reduced by 23% relative to that of the C_3_ species. In proto-Kranz species from other genera, a 5–15% reduction in *Г* has been reported (Sage et al. 2012). Because *Chenopodium* includes numerous proto-Kranz species together with C_3_–C_4_ intermediates, this eudicot genus provides a unique opportunity to elucidate the evolution from C_3_ to proto-Kranz to C_3_–C_4_ intermediate plants.

### Photosynthetic types in *C. album*

We showed that, among *C. album* accessions examined, the accession from Arizona was of the C_3_–C_4_ intermediate type, whereas the remaining six accessions from different localities were of the proto-Kranz type (Table 2). These data suggest that *C. album* may include different photosynthetic types within a species. However, it is well known that *C. album* is a heterogenous assemblage of many taxonomic entities with cosmopolitan distribution, probably because many weedy and semi-domesticated forms have arisen by hybridization and polyploidization (Bhargava et al. 2006; Ohri 2015). It has been recently suggested that hybridization may also be involved in the occurrence of C_3_–C_4_ intermediates (Kadereit et al. 2017; Ueno et al. 2006). Strict genetic and taxonomic studies will be required to ascertain whether different photosynthetic types occur within *C. album*. On the other hand, it cannot be ruled out that environmental factors may influence the expression level of C_3_–C_4_ intermediate traits (Teese 1995). Oono et al. (2017) have recently reported that high growth temperature and low nitrogen level in soil induce a decrease in *Г* and stronger expression of GDC-P in BS cells relative to M cells in the Tsukuba accession of *C. album*. Further research on *C. album* would provide better understanding of the ecological and adaptive aspects and the expression of C_3_–C_4_ intermediate traits.

## Electronic supplementary material

Below is the link to the electronic supplementary material.

Supplementary material 1 (PDF 4276 kb)
