# Correction to: Transition from C_3_ to proto-Kranz to C_3_–C_4_ intermediate type in the genus *Chenopodium* (Chenopodiaceae)

**DOI:** 10.1007/s10265-020-01192-1

**Published:** 2020-04-25

**Authors:** Yuki Yorimitsu, Aya Kadosono, Yuto Hatakeyama, Takayuki Yabiku, Osamu Ueno

**Affiliations:** 1 Graduate School of Bioresource and Bioenvironmental Sciences, Kyushu University, Motooka 744, Nishi‑ku, Fukuoka, 819‑0395 Japan; 2 School of Agriculture, Kyushu University, Motooka 744, Nishi‑ku, Fukuoka, 819‑0395 Japan; 3 Present Address: Faculty of Agriculture, Kyushu University, Motooka 744, Nishi‑ku, Fukuoka, 819‑0395 Japan

## Correction to: Journal of Plant Research (2019) 132:839–855 https://doi.org/10.1007/s10265-019-01135-5

The article Transition from C_3_ to proto-Kranz to C_3_–C_4_ intermediate type in the genus *Chenopodium* (Chenopodiaceae), written by Yuki Yorimitsu, Aya Kadosono, Yuto Hatakeyama, Takayuki Yabiku and Osamu Ueno, was originally published Online First without Open Access. After publication in volume 132, issue 6, page 839–855 the author decided to opt for Open Choice and to make the article an Open Access publication. Therefore, the copyright of the article has been changed to © The Author(s) 2020 and the article is forthwith distributed under the terms of the Creative Commons Attribution 4.0 International License (https://creativecommons.org/licenses/by/4.0/), which permits use, duplication, adaptation, distribution and reproduction in any medium or format, as long as you give appropriate credit to the original author(s) and the source, provide a link to the Creative Commons license, and indicate if changes were made.

The original article was updated.

