# Supplementary material for: Transition from C3 to proto-Kranz to C3–C4 intermediate type in the genus Chenopodium (Chenopodiaceae)
Source: J Plant Res. 2019 Aug 31;132(6):839–55. doi: 10.1007/s10265-019-01135-5 (PMC7205854; doi:10.1007/s10265-019-01135-5)
Supplement: Supplementary file 1 — Supplementary material 1 (PDF 4276 kb) [file 10265_2019_1135_MOESM1_ESM.pdf]

Electronic supplementary materials

Title:

Transition from C_3 to proto-Kranz to C_3–C_4 intermediate type in the genus *Chenopodium* (Chenopodiaceae)

Authors:

Yuki Yorimitsu, Aya Kadosono, Yuto Hatakeyama, Takayuki Yabiku, Osamu Ueno

Journal:

Journal of Plant Research

Corresponding author:

Osamu Ueno

(Affiliation, Address, Country)

Faculty of Agriculture, Kyushu University, Motooka 744, Nishi-ku, Fukuoka 819-0395, Japan

Fax: +81-92-802-4561

E-mail: uenoos@agr.kyushu-u.ac.jp

Content:

Figs. S1–S9

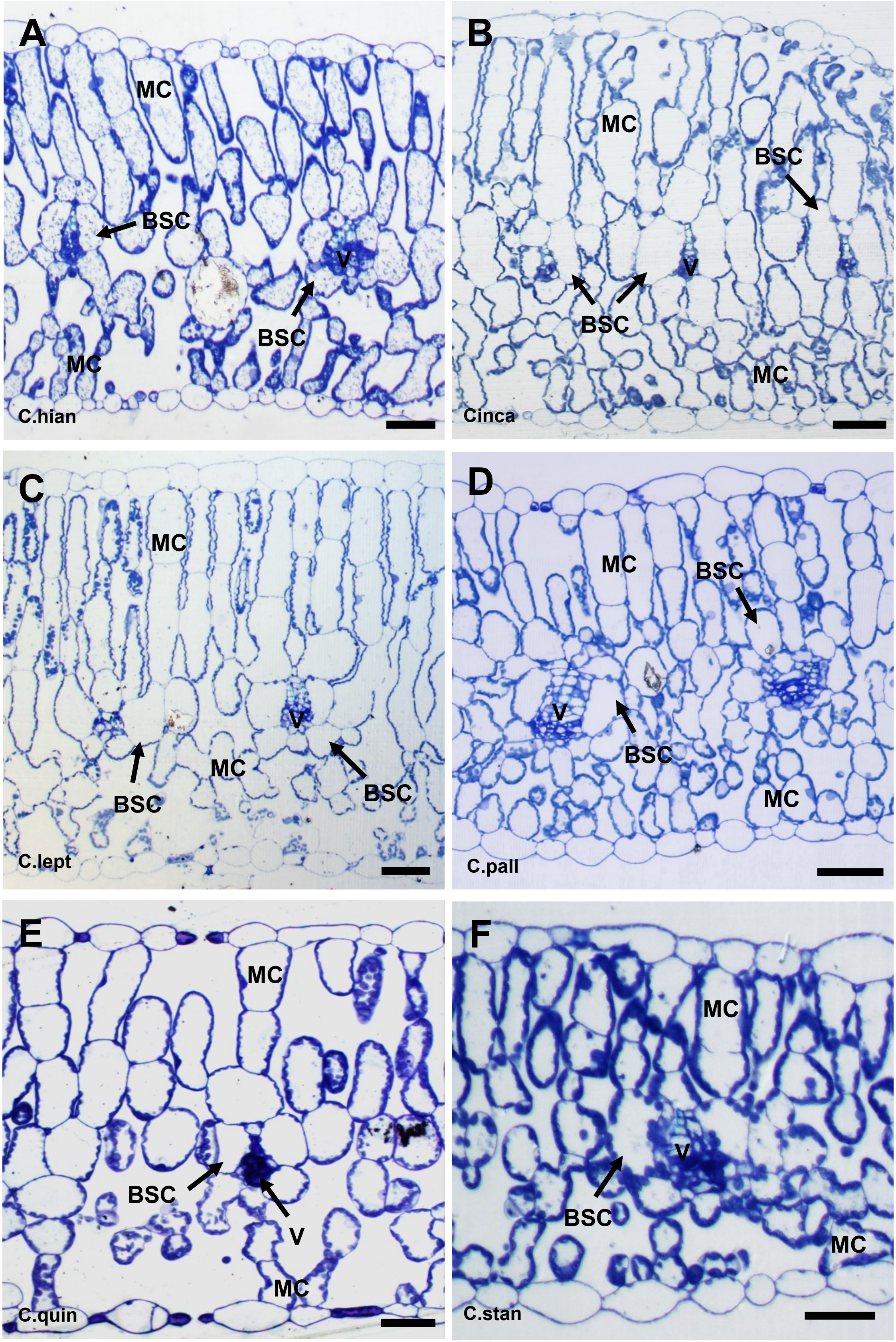

Fig. S1. Leaf structure of *Chenopodium* species with non-Kranz anatomy. (A) *C. hians*; (B) *C. incanum*; (C) *C. leptophyllum*; (D) *C. pallidicaule*; (E) *C. quinoa*; (F) *C. standleyanum*. BSC, bundle-sheath cell; MC, mesophyll cell; V, vascular bundle. Bars = 50 µm.

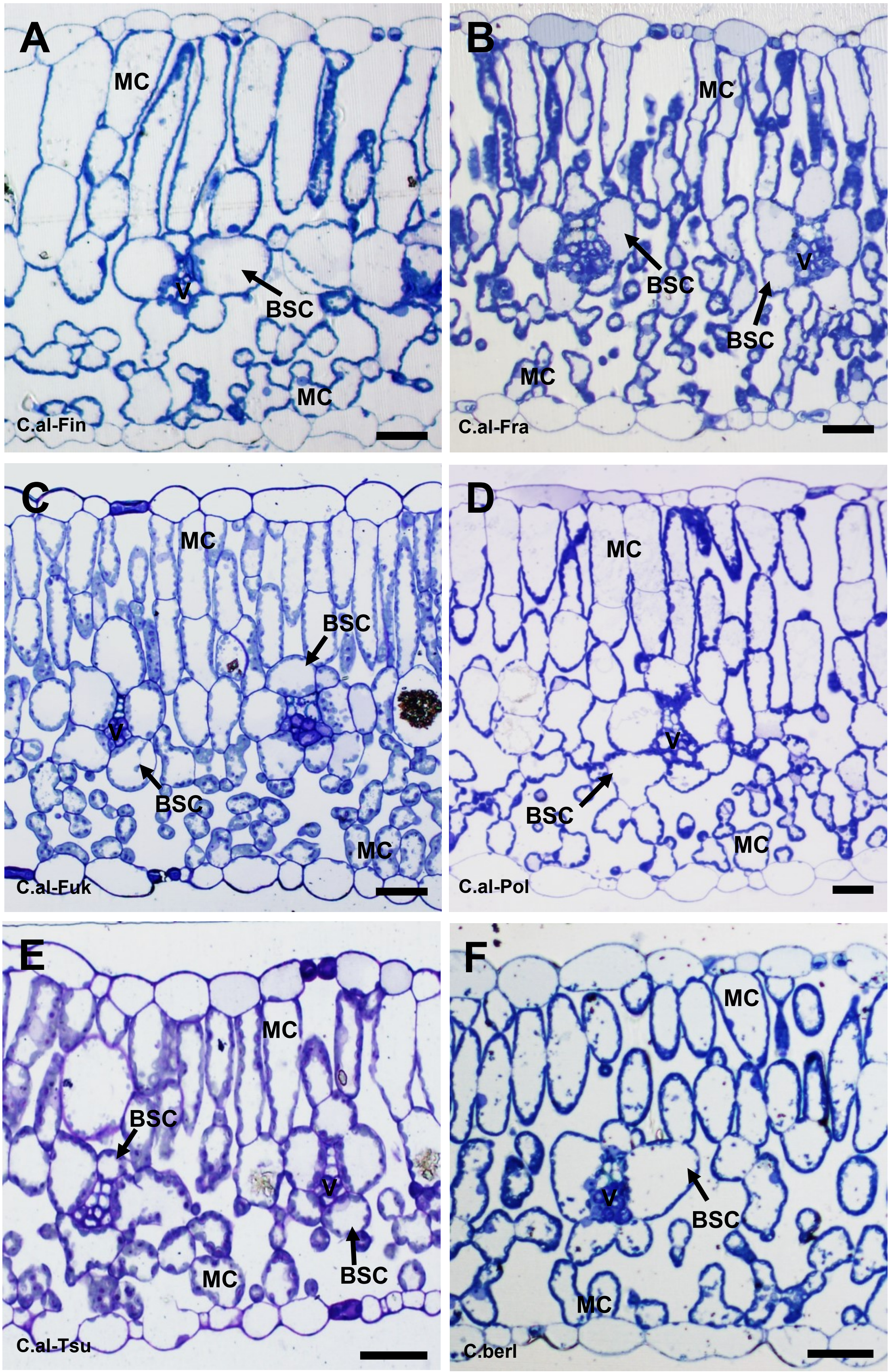

Fig. S2. Leaf structure of *Chenopodium* species with proto-Kranz anatomy. (A) *C. album* (Finland); (B) *C. album* (France); (C) *C. album* (Fukuoka); (D) *C. album* (Poland); (E) *C. album* (Tsukuba); (F) *C. berlandieri*. BSC, bundle-sheath cell; MC, mesophyll cell; V, vascular bundle. Bars = 50 µm.

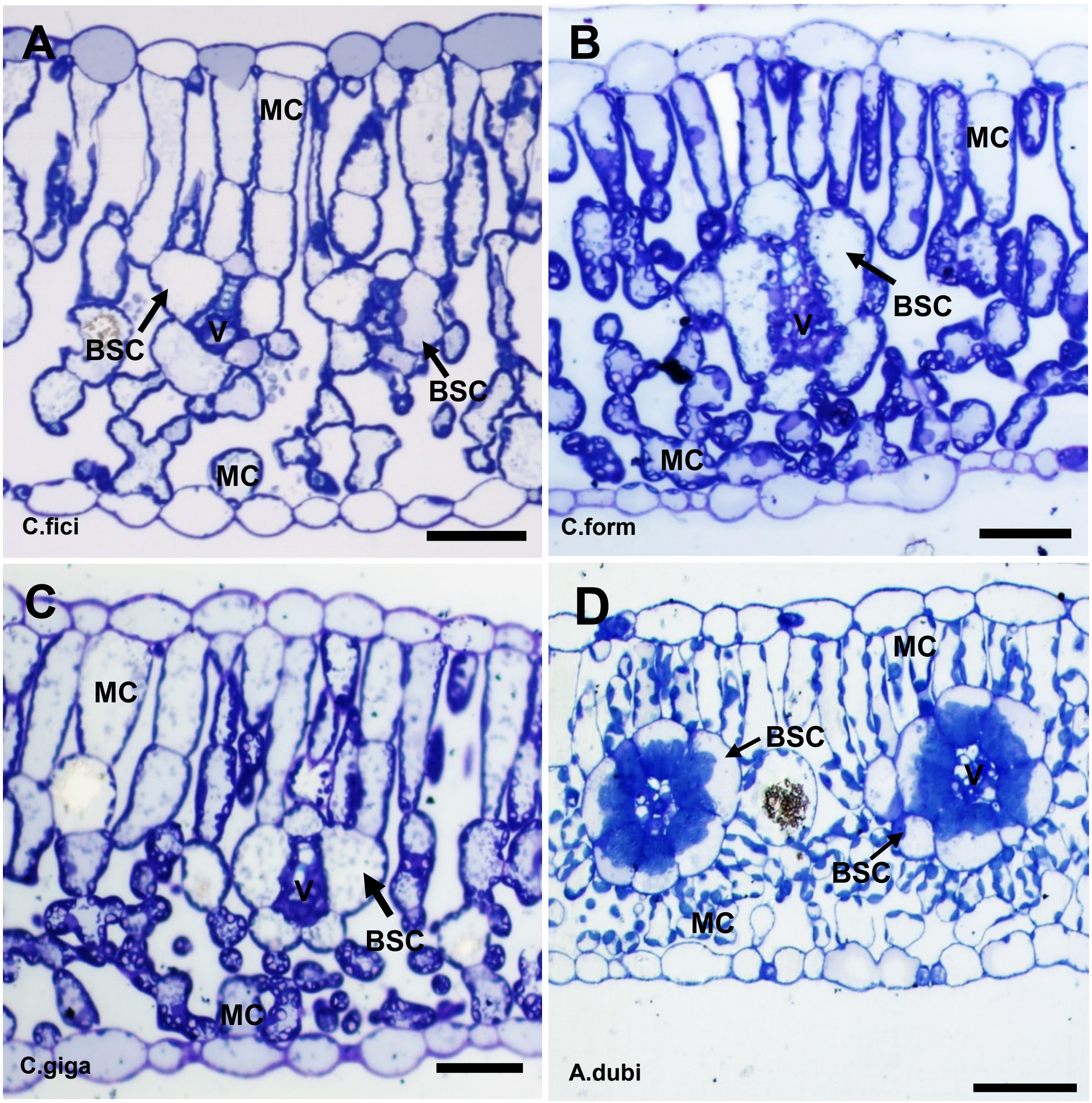

Fig. S3. Leaf structure of *Chenopodium* species with proto-Kranz anatomy (A–C) and *Amaranthus dubius*, which has Kranz anatomy (D, C_4 control). (A) *C. ficifolium*; (B) *C. formosanum*; (C) *C. giganteum*. BSC, bundle-sheath cell; MC, mesophyll cell; V, vascular bundle. Bars = 50 μm.

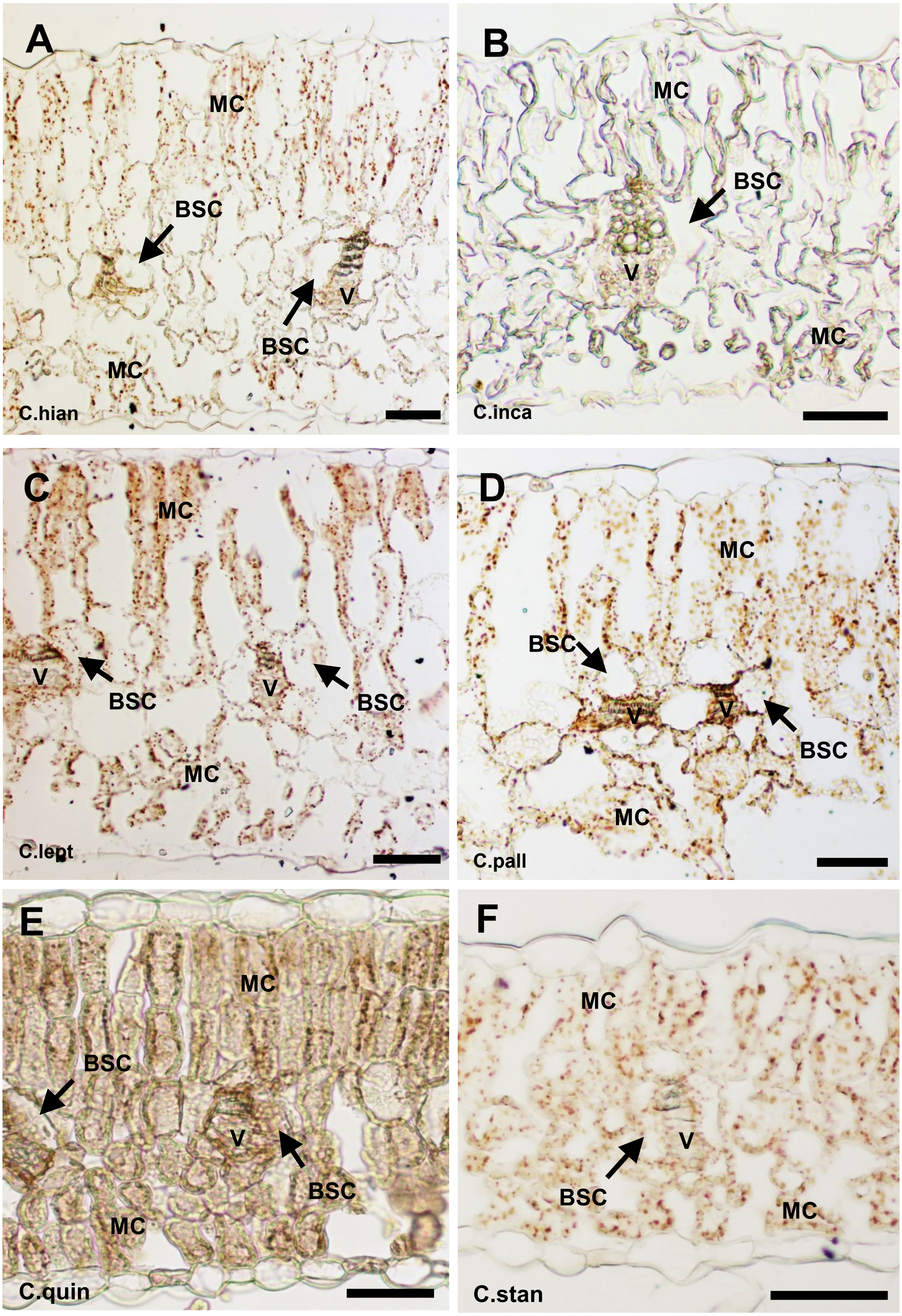

Fig. S4. Immunohistochemical staining of GDC-P in mesophyll and bundle-sheath cells of *Chenopodium* species with non-Kranz anatomy. (A) *C. hians*; (B) *C. incanum*; (C) *C. leptophyllum*; (D) *C. pallidicaule*; (E) *C. quinoa*; (F) *C. standleyanum*. BSC, bundle-sheath cell; MC, mesophyll cell; V, vascular bundle. Bars = 50 µm.

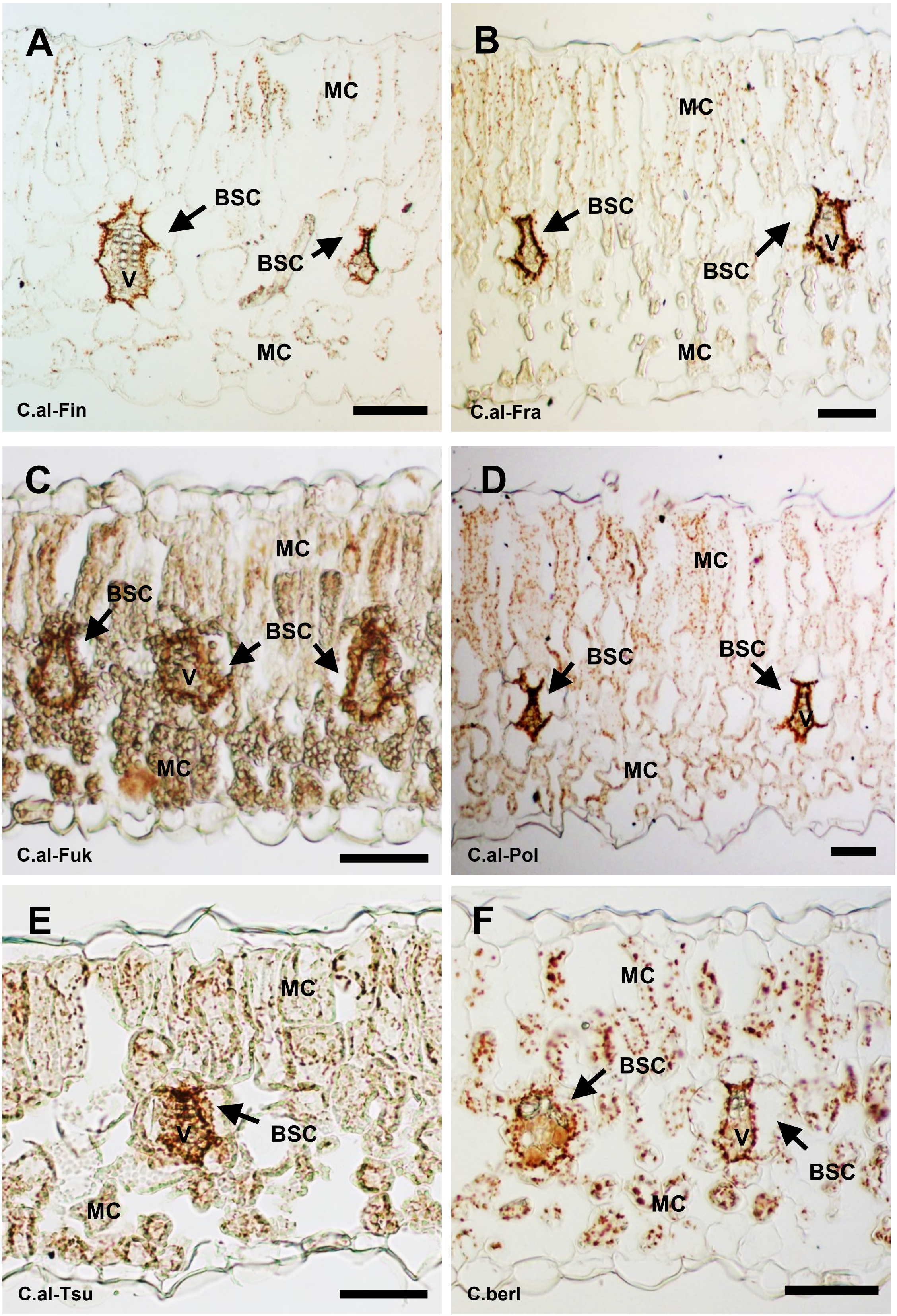

Fig. S5. Immunohistochemical staining of GDC-P in mesophyll and bundle-sheath cells of *Chenopodium* species with proto-Kranz anatomy. (A) *C. album* (Finland); (B) *C. album* (France); (C) *C. album* (Fukuoka); (D) *C. album* (Poland); (E) *C. album* (Tsukuba); (F) *C. berlandieri*. BSC, bundle-sheath cell; MC, mesophyll cell; V, vascular bundle. Bars = 50 µm.

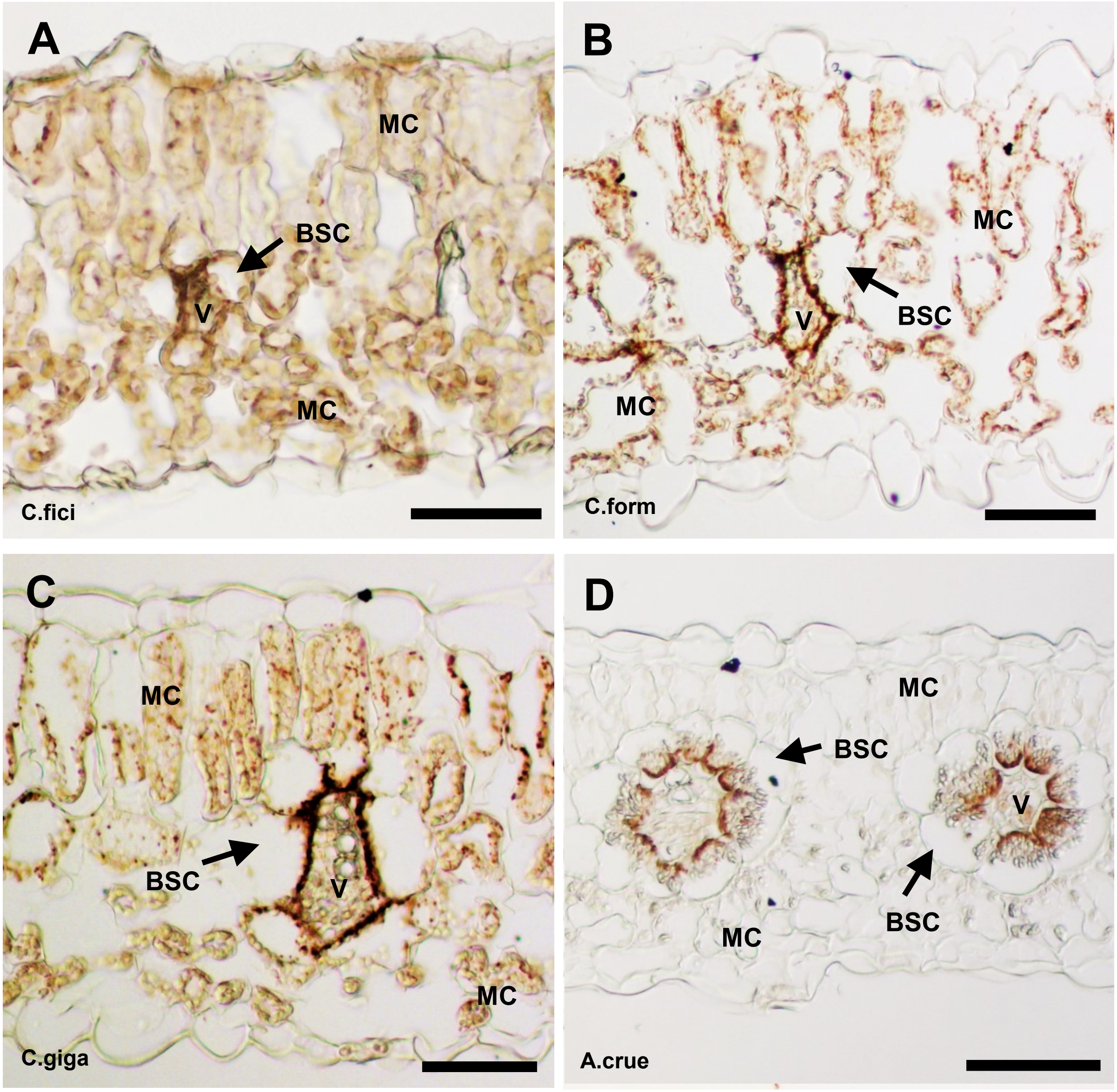

Fig. S6. Immunohistochemical staining of GDC-P in mesophyll and bundle-sheath cells of *Chenopodium* species with proto-Kranz anatomy (A–C) and *Amaranthus cruentus*, which has Kranz anatomy (D, C_4 control). (A) *C. ficifolium*; (B) *C. formosanum*; (C) *C. giganteum*. BSC, bundle-sheath cell; MC, mesophyll cell; V, vascular bundle. Bars = 50 µm.

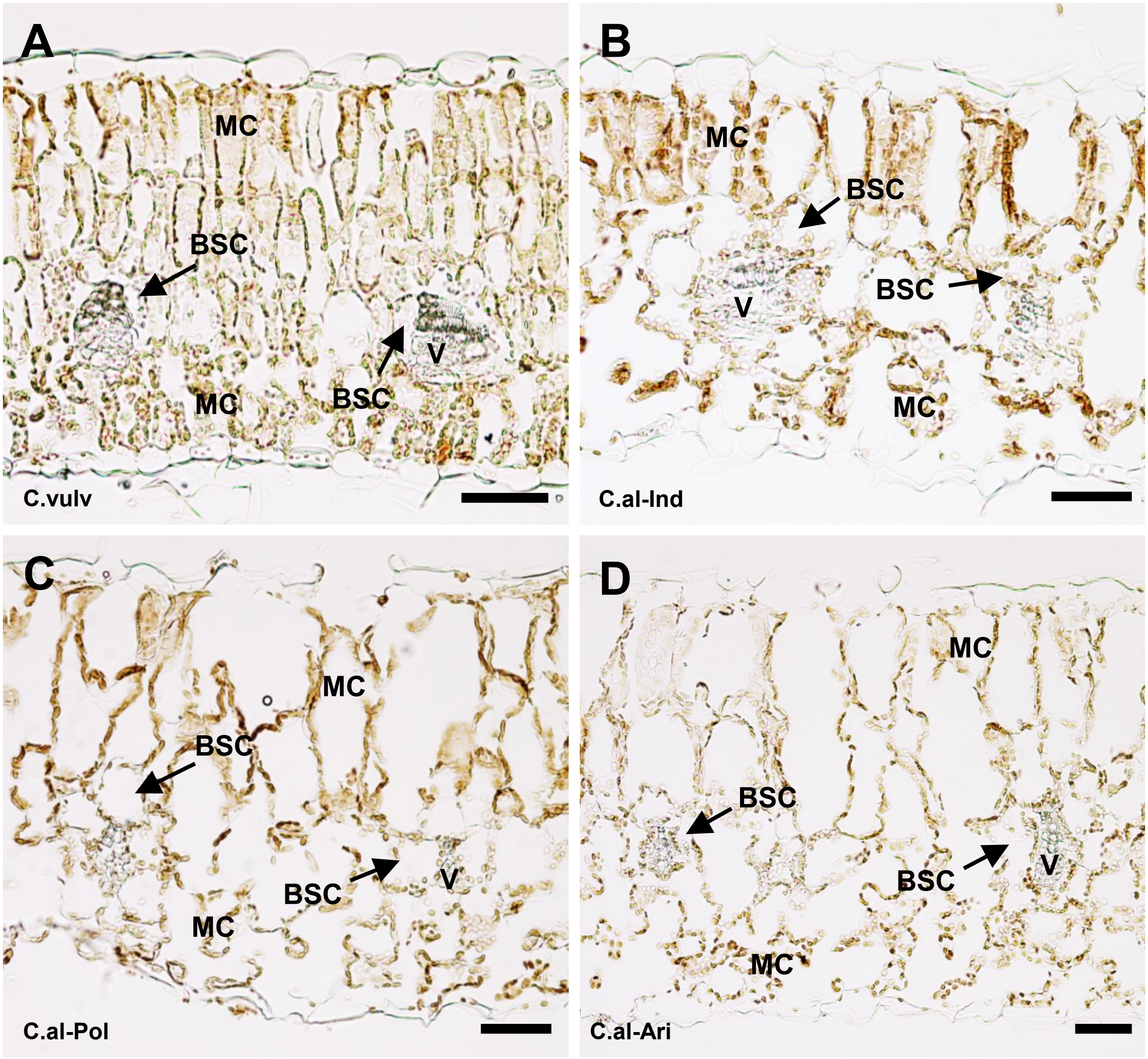

Fig. S7. Immunohistochemical staining of Rubisco LSU in mesophyll and bundle-sheath cells of *Chenopodium* species. (A) *C. vulvaria* (non-Kranz); (B) *C. album* (India, proto-Kranz); (C) *C. album* (Poland, proto-Kranz); (D) *C. album* (Arizona, USA, Kranz-like). BSC, bundle-sheath cell; MC, mesophyll cell; V, vascular bundle. Bars = 50 μm.

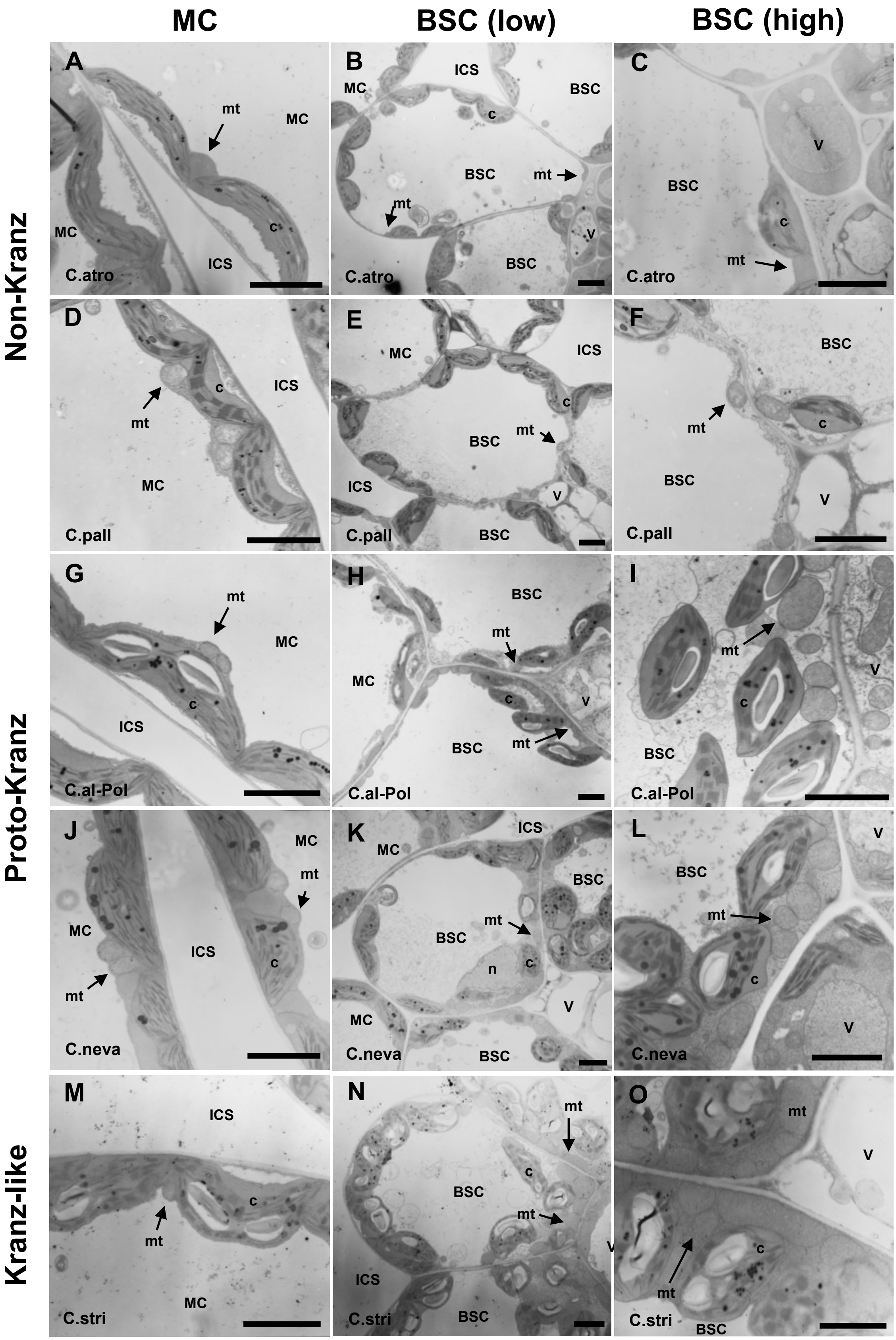

Fig. S8. Ultrastructure of mesophyll cells (A, D, G, J, M) and bundle-sheath cells at a low (B, E, H, K, N) and at a high (C, F, I, L, O) magnification in *Chenopodium* species. (A–C) *C. atrovirens*; (D–F) *C. pallidicaule*; (G–I) *C. album* (Poland); (J–L) *C. nevadense*; (M–O) *C. strictum*. BSC, bundle-sheath cell; ICS, intercellular space; MC, mesophyll cell; V, vascular bundle; c, chloroplast; mt, mitochondrion; n, nucleus. Bars = 3 μm.

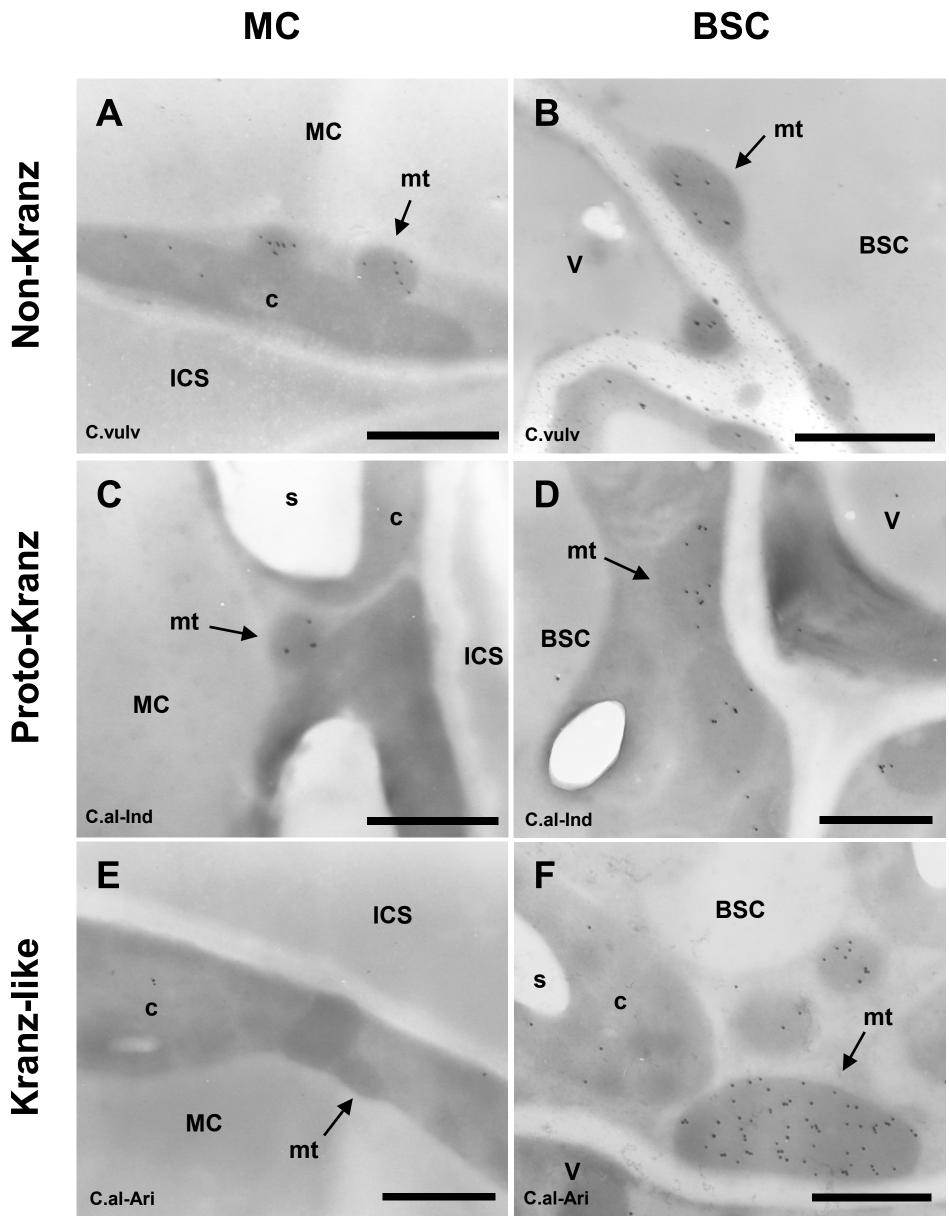

Fig. S9. Immunogold localization of GDC-P in mesophyll (A, C, E) and bundle-sheath cells (B, D, F) cells of *Chenopodium* species. (A, B) *C. vulvaria*; (C, D) *C. album* (India); (E, F) *C. album* (Arizona, USA). BSC, bundle-sheath cell; ICS, intercellular space; MC, mesophyll cell; V, vascular bundle; c, chloroplast; mt, mitochondrion; s, starch grain. Bars = 1 μm.
